# Phylogenetics and evolution of *Su(var)3-9 SET *genes in land plants: rapid diversification in structure and function

**DOI:** 10.1186/1471-2148-11-63

**Published:** 2011-03-09

**Authors:** Xinyu Zhu, Hong Ma, Zhiduan Chen

**Affiliations:** 1 State Key Laboratory of Systematic and Evolutionary Botany, Institute of Botany, Chinese Academy of Sciences, Beijing 100093, China; 2 School of Life Sciences, Nantong University, Nantong 226019, China; 3 State Key Laboratory of Genetic Engineering, School of Life Sciences, Institute of Plant Biology, Center for Evolutionary Biology, Institutes of Biomedical Sciences, Fudan University, Shanghai 200433, China; 4 Department of Biology, the Huck Institutes of the Life Sciences, The Pennsylvania State University, University Park PA 16802, USA

## Abstract

**Background:**

Plants contain numerous *Su(var)3-9 *homologues (*SUVH*) and related (*SUVR*) genes, some of which await functional characterization. Although there have been studies on the evolution of plant *Su(var)3-9 SET *genes, a systematic evolutionary study including major land plant groups has not been reported. Large-scale phylogenetic and evolutionary analyses can help to elucidate the underlying molecular mechanisms and contribute to improve genome annotation.

**Results:**

Putative orthologs of plant Su(var)3-9 SET protein sequences were retrieved from major representatives of land plants. A novel clustering that included most members analyzed, henceforth referred to as core *Su(var)3-9 *homologues and related (*cSUVHR*) gene clade, was identified as well as all orthologous groups previously identified. Our analysis showed that plant Su(var)3-9 SET proteins possessed a variety of domain organizations, and can be classified into five types and ten subtypes. Plant *Su(var)3-9 SET *genes also exhibit a wide range of gene structures among different paralogs within a family, even in the regions encoding conserved PreSET and SET domains. We also found that the majority of SUVH members were intronless and formed three subclades within the SUVH clade.

**Conclusions:**

A detailed phylogenetic analysis of the plant *Su(var)3-9 SET g*enes was performed. A novel deep phylogenetic relationship including most plant *Su(var)3-9 SET *genes was identified. Additional domains such as SAR, ZnF_C2H2 and WIYLD were early integrated into primordial PreSET/SET/PostSET domain organization. At least three classes of gene structures had been formed before the divergence of *Physcomitrella patens *(moss) from other land plants. One or multiple retroposition events might have occurred among *SUVH *genes with the donor genes leading to the V-2 orthologous group. The structural differences among evolutionary groups of plant *Su(var)3-9 SET *genes with different functions were described, contributing to the design of further experimental studies.

## Background

The SET domain (SM00317) is the catalytic center of lysine methyltransferases with a conserved sequence of ~130 amino acid residues, initially identified at the C- terminus of three regulatory factors (Su (var)3-9, E(z) and Trithorax) in *Drosophila* accounting for its name [1-4]. Currently, proteins containing the conserved SET domain can be found in organisms ranging from virus to all three domains of life (Bacteria, Archaea, and Eukaryota) [5]. In plants, Baumbusch et al. [6] first identified 37 putative *Arabidopsis *SET genes, and divided them into four distinct classes: (1) E(Z) homologues; (2) Ash1 homologues and related genes; (3) trx homologues and related genes; and (4) Su(var) homologues and related genes. Subsequently, Springer et al. [7] added 25 maize SET genes to those of 37 *Arabidopsis*, and divided them into five classes based on phylogenetic relationships and domain organization; among these, the Su(var) homologues and related genes were designated as class V. Recently, Ng et al. [8] established two additional plant SET-gene classes, i.e. class VI composed of the SET genes [9] and VII composed of the Putative RuBisCo genes [10]; however, these recent classes lack typical SET domain, either interrupted in the SET-I region of SET domain or truncated.

Among the seven classes of plant SET genes, class V contains significantly more members relative to other classes and possess the PreSET domain (SM00468) in their proteins [7,8]; for example, from class I to VII, *Arabidopsis *contains 3, 5, 7, 2, 15, 5, and 9 members, respectively. Numerous copies in class V may complicate the evolutionary process of this class of plant SET genes. Previous studies [6,7,11] demonstrated that the class V SET proteins can be further divided into seven orthologous groups (V-1 to 7) and two major types (i.e. SUVH and SUVR) based on their phylogenetic relationship and domain organization. The SUVH proteins consist of orthologous groups V-1, 2, 3 and 5, and have an additional evolutionarily conserved SRA domain (SM00466) upstream of the PreSET domain. The SUVR proteins are composed of the remaining V-4, 6 and 7 orthologous groups and lack the SRA domain. Baumbusch et al. [6] and Springer et al. [7] noted that the majority of SUVH members in *Arabidopsis *and maize lacked introns, and supposed that these intronless SUVH members probably originated from ancient retrotransposition events.

In *Arabidopsis*, there are ten *SUVH *and five *SUVR *genes, in which five *SUVHs *and three *SUVRs *have been characterized functionally [[12], and references therein]. SUVH1, 2, 4, 5 and 6 have been shown to control heterochromatic silencing by the HMTase activity [13-17], and SUVR1, 2, 4 were mainly localized in the nucleolus or nuclear bodies, suggestive of involvement in regulation of rRNA expression [12]. In contrast to SUVH proteins, SUVR4 acts as a dimethyltransferase with preference for mono-methylated H3K9 as substrate, suggesting that SUVHs and SUVRs can act in concert in achieving various functional H3K9 methylation states. It has also been found that the SRA domain of the SUVH proteins may be involved in heterochromatin formation mediated by H3K9 methylation [16]. SUVRs, however, were once supposed to lack a shared N-terminal domain, although a novel conserved N-terminal domain, WIYLD (PF10440), was recently identified in a few members of the V-6 orthologous group, such as the *Arabidopsis *SUVR1, 2, and 4 [12].

Here, we sampled from ten representatives of land plants to investigate the phylogeny and evolution of plant *Su(var)3-9 SET* genes. This is the first analysis of these genes covering the range of land plants. We performed phylogenetic analysis using the combined datasets from the sequences of the conserved PreSET- and SET-domain regions to increase phylogenetic resolution. On the basis of phylogenetic analyses, we tracked the evolution of domain organizations and gene structures of plant *Su(var)3-9 SET* genes in land plants; in turn, these domain organizations and gene structures were used as synapomorphies (derived character states shared by two or more taxa/members) to confirm the phylogenetic relationships. Finally, we explored the relationships between evolutionary patterns and functional diversification by combining the phylogenetic results with available literature for functions of plant *Su(var)3-9 SET* genes; the results of our study would lay the foundation for the design of future experimental studies.

## Results

### Plant *Su(var)3-9 SET *genes

*Arabidopsis thaliana *and *Oryza sativa *contained 15 and 12 full-length Su(var)3-9 SET protein sequences, respectively. To undertake an evolutionary analysis of *Su(var)3-9 SET *genes in land plants, three other completely sequencing plant genomes and one algal genome were searched using multiple representatives of Su(var)3-9 SET proteins in *Arabidopsis thaliana *as queries. By conducting tBLASTn searches against the JGI genome database, we obtained 16, 5, 7 and 1 Su(var)3-9 SET protein sequences from *Populus trichocarpa *(Pt), *Selaginella moellendorfii *(Sm), *Physcomitrella patens *(Pp) and *Chlamydomonas reinhardtii *(Cr), respectively. Seven cDNA sequences of *Pinus taeda *(Pta) were obtained from TIGR plant indices. In addition, 1, 2 and 7 Su(var)3-9 SET protein sequences were also obtained from *Nicotiana tabacum *(Nt), *Vitis vinifera *(Vv) and *Ricinus communis *(Rc), respectively. In total, 74 candidate SET sequences were collected from ten species, and the detailed information is provided in Additional file 1 and 2. Protein sequences lacking PreSET domain were not used for the further study even when they have very low *E *values in the BLAST searches. The *Arabidopsis *SDG11 (SUVH10) was also not used because it is likely a pseudogene [6].

### Phylogenetic analysis

**Figure 1:**
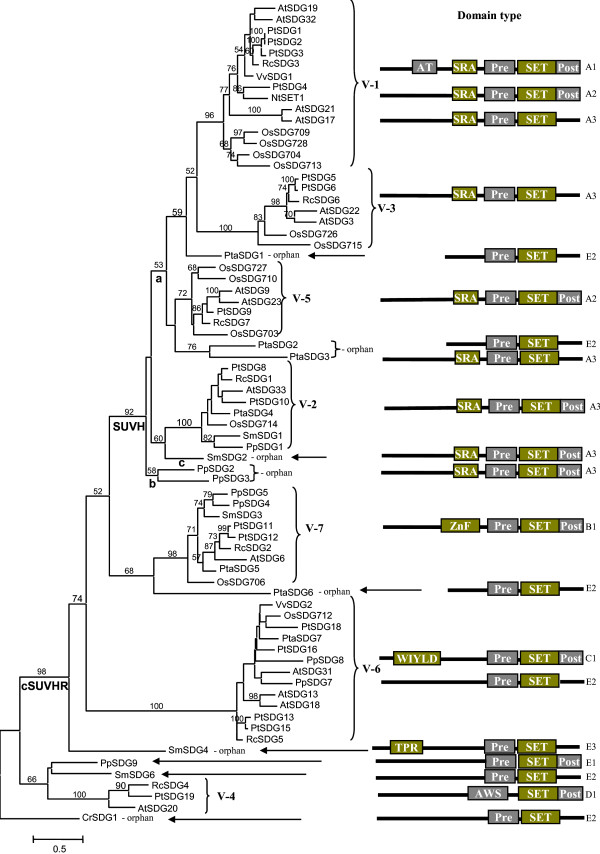
**An ML phylogenetic tree of plant Su(var)3-9 SET proteins**. The numbers above branches are bootstrap percentages >50, and those below are the clade name. The lowercase letter "a, b, c" represent three intronless clades. The name of Su(var)3-9 SET protein sequences is formed through species abbreviation plus SDG (SET-domain protein group) numbering. Species abbreviation: At, *Arabidopsis thaliana*; Os, *Oryza sativa*; Pt, *Populus trichocarpa*; Nt, *Nicotiana tabacum*; Vv, *Vitis vinifera*; Rc, *Ricinus communis*; Pta, *Pinus taeda*; Sm, *Selaginella moellendorfii*; Pp, *Physcomitrella patens*; Cr, *Chlamydomonas reinhardtii*. The SDG numbering for *Arabidopsis thaliana *and *Oryza sativa *are from ChromDB (http://www.chromdb.org/), and these of other species are numbered in this study. Domain type (see Table 1) within each corresponding clade is depicted on the right. Domain abbreviations: AT, AT_hook; Pre, PreSET; Post, PostSET; ZnF, ZnF_C2H2; TPR, TPR_1.

Alignment of the combined dataset from PreSET and SET domains resulted in a matrix with length of 228 sites after removing ambiguous regions and autapomorphic insertions (see Additional file 3). The WAG model [18] was selected as the best-fit evolutionary model under the AIC criterion [19] with specific improvements (+G [20]; +F [21]). A maximum-likelihood (ML) analysis produced an optimal tree with an InL score of -20557.59. The NJ analyses recovered trees with almost identical topologies and support values to those of ML analyses. Most of differences between ML and NJ trees were distributed on extremely short branches (see Additional file 4). The ML tree is presented in Figure 1 with bootstrap percentages at the node of the branch.

**Figure 2:**
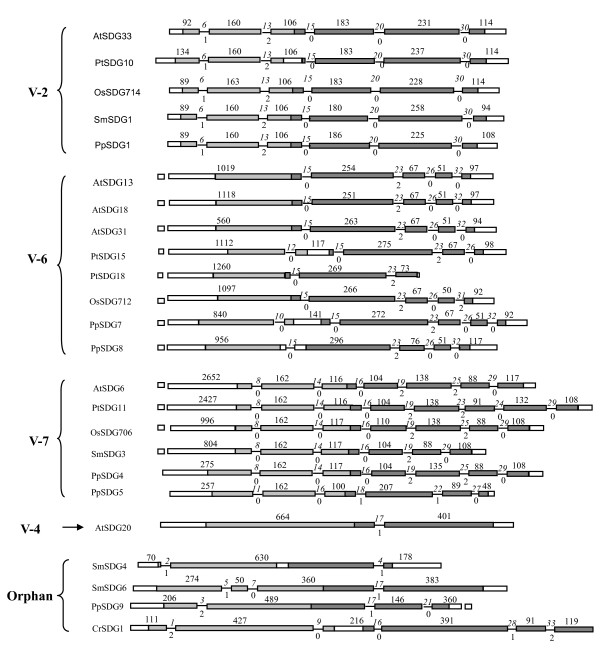
**Schematic representation of the *Su(var)3-9 SET *gene structures in PreSET and SET domain regions**. Boxes represent exons and lines represent introns. Length of exons are roughly at scale but introns not. Dark gray regions encode for SET domains; light gray regions encode for PreSET domains. The numbers above boxes are the length of exons (bp). The numbers above lines are the intron positions (see Additional file 5 for the alignment of mRNA sequences) and those below are the intron phases.

Our analysis recovered all orthologous groups previously identified [6,7,11] (Figure 1). In the present investigation, we broadened these orthologous groups (group (s), hereafter) based on internal support (>95% BS) or conserved domain organization and gene structure (Table 1 and Figure 2), thus resulting in the inclusion of more members in each group. In the current study, we used the definition of groups previously identified [6,7,11] mainly for the purpose of comparison, and it is possible that some groups we have designated as a single group might actually represent multiple groups because of sampling limitations. Our tree showed that all members could be divided into two clades when the member of *Chlamydomonas reinhardtii *(green alga) was designed as the outgroup. The smaller clade was moderately supported, including V-4 group and other two members; it is worth noting that the V-4 group only contained angiosperm members excluding rice (monocot); the larger clade was strongly supported, including all the remaining members, which was named as cSUVHR (core Su(var)3-9 homologues &related genes) clade in our analysis (Figure 1). Within the cSUVHR clade, the subclade including V-1, 2, 3, and 5 groups was strongly supported and named here as the SUVH clade (Figure 1) because all members possess a characteristic SRA domain at the N terminus [7]; this result was consistent with a previous hypothesis that all *SUVH *genes had a common ancestor [7,11]. The V-7 group within the cSUVHR clade appears to be sister to the SUVH clade, but only with low support. The V-6 group within the cSUVHR clade was placed at the basal position that did not appear to have a clear relationship with other groups.

**Table 1:** The domain organizations of plant Su(var)3-9 SET proteins.

Type	Subtype	Domain architectures	Species										Distribution
				
			At	Os	Pt	Nt	Rc	Vv	**Pt**a	Sm	Pp	Cr	
A	1	AT_hook-SRA-PreSET-SET-PostSET	+	+	-	-	-	-	-	-	-	-	V1
	2	XXX-SRA-PreSET-SET-PostSET	+	+	+	+	+	+	+	+	+	-	V1,V2,V5
	3	XXX-SRA-PreSET-SET-XXX	+	+	+	-	+	-	+	-	-	-	V1,V2,V3
B	1	ZnF_C2H2-PreSET-SET-PostSET	+	+	+	-	+	-	-	+	+	-	V7
C	1	WIYLD-PreSET-SET-PostSET	+	+	+	-	-	-	-	-	+	-	V6
	2	WIYLD-PreSET-SET-XXX	+	-	+	-	+	+	-	-	-	-	V6
D	1	AWS-SET-PostSET	+	-	+	-	+	-	-	-	-	-	V4
E	1	XXX-PreSET-SET-PostSET	-	-	+	-	-	-	-	-	+	-	Orphan
	2	XXX-PreSET-SET-XXX	-	-	+	-	+	-	+	-	+	+	V6, Orphan
	3	TPR-PreSET-SET-XXX	-	-	-	-	-	-	-	+	-	-	Orphan

Plus sign (+) and minus sign (-) indicate presence and absence of a subtype, respectively; domain and species abbreviations are listed in figure 1; XXX indicates the protein sequence regions without predicted domain.

Within the SUVH clade, the V-1, V-3 and V-5 groups plus several orphan members (PtaSDG1, 2 and 3) formed a subclade with low support and only seed plant members; in contrast, the V-2 group was strongly supported and contained all representative land plants, with usually one copy in each species (Figure 1); the SUVH clade also contained several other orphan members (SmSDG2, PpSDG2 and PpSDG3), and their relationships with other members of this clade were uncertain (Figure 1). The V-6 group contained members from both seed plants and moss, but not *Selaginella moellendorfii *(fern), and was characterized by the WIYLD domain at the N-terminal region of protein sequences [12]. The V-7 group contained members from each major land plant groups with one or several copies in each species, and possessed a characteristic ZnF_C2H2 domain (SM00355) [22] at the N-terminal region of protein sequences. An orphan member SmSDG4 possessed a unique TPR_1 domain (PF00515) at the N-terminal regions of its protein sequences.

### Domain organization

To trace their evolutionary history in land plants, we predicted the domain organization of candidate Su(var)3-9 SET proteins. The candidate proteins could be classified into five types (groups) and ten subtypes (subgroups) based on their domain organization, with the major differences lying in their N-terminal regions. Type A contained a characteristic SRA domain at the N-terminus (Table 1), which was identified as the YDG_SRA domain (PF02182) in the Pfam platform [6]. The subtype A1, which only existed in V-1 group, had an additional N-terminal domain, AT_hook (SM00348), a small DNA-binding motif that functions in the transcription regulation of genes containing or in close proximity to AT-rich regions [23,24]. In contrast, the subtypes A2 and A3 were broadly distributed in V-1, 2, and 3 and V-5 groups. It was also worth noting that all members in V-3 group lack the PostSET domain (SM00508) at their C-terminal regions. Type B contains one or more ZnF_C2H2 domain(s) at its N-terminus (Table 2) and was only distributed in the V-7 group. The ZnF_C2H2 domain is one type of the C2H2-type zinc fingers (Znf), very common DNA-binding motifs found extensively in eukaryotic and prokaryotic transcription factors [25,26]. Type C contains one WIYLD domain at its N-terminus and was only found in the V-6 group (Table 2). Type D lacked a typical PreSET domain and contains instead the AWS domain (SM00570) (Associated With SET), a subdomain of PreSET domain. This domain organization might have arisen recently because it was only found in angiosperms in the present study. The AWS domain was often found in association with the SET domain, suggesting a role in methylation of lysine residues in histones and other proteins [27]. Type E refers to remaining domain organizations that were mostly from orphan members, either lacking identifiable N-terminal domains or having unique N-terminal domains; subtype E1 and E2 might be the ancestral domain organization due to their extensive distribution in eukaryotes (data not shown).

**Table 2:** Phase and number of introns in plant *Su(var)3-9 SET *genes

	No. of introns in each phase (%)				
			
Clade (no. of genes)	0	1	2	Total no. of introns	Mean no. of introns per gene
V-2 (5)	43	14	14	71	14
V-4 (1)	0	1	0	1	1
V-6 (8)	35	12	12	59	7.6
V-7 (6)	39	9	9	57	9.5
Orphan (4)	8	7	5	20	5
Total (24)	125(61)	43(20)	40(19)	208	8.7

### Gene structure

In the present study, the structures of only 24 plant *Su(var)3-9 SET *genes (see Additional file 5 and 6) were analyzed due to the lack of the corresponding genomic sequences in other *Su(var)3-9 SET *genes. We found that the number of intron is highly variable in plant *Su(var)3-9 SET *genes, ranging from 0 in V-1, -3, and -5 groups to 20 in SmSDG1. A total of 208 introns were present in 24 analyzed genes, an average of 8.7 introns per gene; the average number of introns per gene also varied among groups, ranging from 7.6 in V-6 group to 14 in V-2 group (Table 2). Among the 208 introns, 125 (61%) were in phase 0, 43 (20%) in phase 1, and 40 (19%) in phase 2 (Table 2), similar to the previous reports of 57.3% for phase 0, 21.5% for phase 1, and 21.2% for phase 2 in 21,570 rice genes [28]. To trace the evolutionary pattern of gene structure, the current study mainly focused on the most conserved PreSET and SET domain regions. Figure 2 presents the gene structures of these two regions. Our result showed that at least three classes of gene structures (i.e. V-2, V-6 and V-7 groups) were formed probably through frequent inron loss and gain before the divergence of *Physcomitrella patens *from other land plants. In these three groups, the ancestral gene structures might be similar to PpSDG1, PpSDG8 and PpSDG4 (Figure 2), respectively. The sequence similarity between introns was not analyzed because their lengths were highly variable. Within the V-2 group, all introns maintained identical phases and positions, indicating a high degree of structural conservation during the evolution of land plants. In contrast, the V-6 and -7 groups were less conserved; for example, in V-6 the intron sliding occurred in the last intron (position 31 of OsSDG712) (see Additional file 5). Also in V-7 PpSDG5 had only one conserved intron (position 16) compared to other genes. AtSDG20, SmSDG6 and PpSDG9 had a common intron (position17), together with the low support on the relationship among them in phylogenetic tree, suggesting that V-4 group might have a common ancestor with these two orphan members.

Most members in the SUVH clade were intronless except for the V-2 group. Previous studies found that most *Arabidopsis *genes in this clade were intronless, and suggested that these intronless members may have originated from one or a few retroposition events, followed by tandem duplication [6]. If this hypothesis is correct, the donor genes of the retroposition might also be in the V-2 group, because the descendent retrogene and the donor genes should cluster together in the phylogenetic tree just as in Figure 1. In the SUVH clade, intronless members formed three independent subclades, each with weak BP support values (see a, b and c branches in Figure 1). If multiple retroposition events occurred in donor gene lineage, the donor gene lineage would cluster with these retrogenes arranged paraphyletically in phylogenetic tree (Figure 1). Owing to the low support values in the current data, we are still unable to determine whether these three intronless branches originated independently or had a common ancestor.

## Discussion

**Figure 3:**
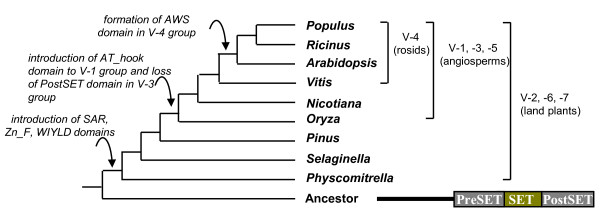
**The proposed evolutionary events of the domain structures of Su(var)3-9 SET proteins in land plant lineages**. Proposed evolutionary events are in italic.

The presence of gene families is one of the characteristics of eukaryotes [29,30]. Since the genes within families are initially redundant in molecular function, they likely have undergone evolutionary selection processes, and eventually formed multiple orthologous groups to carry out different functions [31,32]. The current research first presented the phylogeny and evolution of plant *Su(var)3-9 SET *gene family in land plants. Our analyses identified a novel phylogenetic relationship, that is, the cSUVHR clade that includes most members analyzed except for the V-4 group and a few orphan members (Figure 1). In addition, our results support the following evolutionary scenario of this gene family: multiple gene duplications had occurred independently before the split of *Physcomitrella patens *(moss) from other land plants, and since then each of orthologs experienced molecular divergence by mutations, domain acquisition and gene structure changes, resulted in different orthologous groups. We suggested that the SAR, ZnF_C2H2 and WIYLD domains were early integrated into primordial PreSET/SET/PostSET domain organization to form different evolutionary groups (Figure 3) because the type A, B and C domain organization in Table 1 were all found in *Physcomitrella patens*. In contrast to previous reports [7,8], our analyses showed that the PostSET domain was present in most plant Su(var)3-9 SET proteins, but not in the V-3 group. In the light of the parsimony rule of evolution, we propose that the ancestral plant Su(var)3-9 SET proteins might have possessed the PostSET domain, which was lost in some members during the subsequent evolution (Figure 3).

The plant *Su(var)3-9 *SET gene family exhibits a large diversity of gene structures, even in the conserved PreSET and SET domains (Figure 2), implying frequent gain and/or loss of introns during evolution [33]. For plant *Su(var)3-9 *SET gene family, such frequent gain/loss of introns might have occurred during early evolution of land plants because the gene structure of the V-2, 6 and 7 groups had appeared before the divergence of *Physcomitrella patens *(Figure 2). Because introns might have regulatory functions [34,35], the gain or loss of introns may have contributed to functional divergence between paralogs, such as subfunctionalization, either directly by introducing regulatory differences or by facilitating exon shuffling. In our study, the V-2 group demonstrated strict conservation of gene structure, indicating that this group may have evolved under high selective pressures and is functionally important; in contrast, V-6 and V-7 may have evolved under relatively relaxed selective pressures (Figure 2). As in previous studies [33,36,37], our study showed that the shared variations in gene structure can be used for the classification of paralogous genes into different evolutionary groups (V-2, 6 and 7); accordingly we further suggest that V-4 group be expanded to include two orphan members, SmSDG6 and PpSDG9, because these two genes have a common intron (position17) with AtSDG20 of V-4 group (Figure 2).

In the SUVH clade (Figure 1), the majority of the genes, except the V-2 group, were intronless. We suppose that their last common ancestor might possess introns and the intronless genes (V-1, 3, 5 groups) originated from the lineages leading to the V-2 group by retroposition because all 24 genes analyzed including from basal evolutionary groups (V-4, 6 and 7) and an outgroup gene (CrSDG1) from *Chlamydomonas reinhardtii *(green alga), also possessed introns (Figure 2). Many retrogenes have been identified in plant gene families [31,38-41]. It is generally believed that most retrogenes become non-functional because they lack the regulatory elements required for expression [42]. However, several recent studies have demonstrated that functional genes can occasionally be generated from retrogenes and that these processed genes take on a non-redundant functional role [38-40]. In the plant *Su(var)3-9 SET *gene family, transcripts of many intronless *Arabidopsis*, *Oryza *and maize genes have been detected in RT-PCR and/or microarray analyses [6,7,43], suggesting that some retrogenes of the *Su(var)3-9 SET *family might have gained regulatory elements and became functional.

We found that *Arabidopsis Su(var)3-9 SET *genes of different groups or clades have different functions (Table 3), suggesting that they interact with the different substrates. SUVH4 (SDG33) (also known as KYP [13]) and SUVH2 [16,44] play major roles in *Arabidopsis *histone H3K9 methylation modification; in contrast, the loss of SUVH1 (SDG32) [16] and SUVH5 (SDG9) [17] or SUVH6 (SDG23)[45] results in only minor reductions in global H3K9 methylation levels. SUVR1 (SDG13), SUVR2 (SDG18) and SUVR4 (SDG31) proteins have been studied in detail [12]. They are localized to the nucleolus or non-condensed nuclear bodies, which differs from SUVH proteins localizing to heterochromatin region. *In vitro *SUVR4 acts as efficient dimethyltransferase specifically adding the second methyl group to monomethylated H3K9; in contrast, *in vitro *SUVH4 (SDG33), SUVH5 (SDG9) and SUVH6 (SDG23) proteins are very efficient monomethyltransferases but moderately efficient dimethyltransferase [14,17]. The localization of the SUVR proteins suggests that these proteins are not involved in heterochromatic gene silencing, and may function as a repressor of rDNA gene clusters in the decondensed part of the nucleolus. The *SUVR5 *(SDG6) and *SUVR3 *(SDG20) genes were the only *Arabidopsis *representative of *Su(var)3-9 SET *genes in the V-7 and V-4 groups (Figure 1), respectively, and their functions are unknown and will need to be investigated in the future.

**Table 3:** Orthology groups and functions of *Arabidopsis Su(var)3-9 SET *genes

Orghology groups		Gene name	Function(s)	Refs
*SUVH*	V-1	*Suvh1,3,7,8,10*	heterochromatic silencing (minor roles); monomethyltransferase.	[16]
	V-2	*Suvh4*	heterochromatic silencing (major roles); monodimethyltransferase.	[13,14]
	V-3	*Suvh2,9*	heterochromatic silencing (major roles); monodimethyltransferase.	[16,44]
	V-5	*Suvh5,6*	heterochromatic silencing (minor roles); monodimethyltransferase.	[17,45]
*SUVR*	V-6	*Suvr1,2,4*	nucleolus; repressor of rDNA gene clusters; dimethyltransferase.	[12]
	V-7	*Suvr5*	unknow function.	none
	V-4	*Suvr3*	unknow function.	none

## Conclusions

Our study provides novel phylogenetic relationship and new insights into the evolution of plant *Su(var)3-9 SET *gene family in land plants, which includes most members analyzed except for the V-4 group and a few orphan members. We found that the PostSET is not a common domain in plant Su(var)3-9 SET proteins; it might be an ancestral characteristic of this gene family, which was lost in some members during the evolution. We propose that the SAR, ZnF_C2H2 and WIYLD domains were integrated into primordial domain organization, PreSET/SET/PostSET, during the early evolution of land plant and resulted in evolutionary differentiation. Plant *Su(var)3-9 SET *genes exhibit a diversity of structures, even in the conserved PreSET and SET domain regions. At least three classes of gene structures in the V-2, V-6 and V-7 groups had appeared before the divergence of *Physcomitrella patens *from other land plants through frequent inron loss and gain. In the SUVH clade, the majority of the members were intronless retrogenes, probably originated from the ancestral genes leading to V-2 group with introns. Our results revealed the structural differences among evolutionary groups of plant *Su(var)3-9 SET *genes with different functions, and further predicted that the function of *Arabidopsis SUVR5 *(SDG6) and *SUVR3 *(SDG20) genes belonging to the V-7 and V-4 groups, respectively, are different from other *Arabidopsis Su(var)3-9 SET *genes.

## Methods

### Homologous Su(var)3-9 SET proteins search

Six completely sequenced plant genomes were selected for retrieving the Su(var)3-9 SET protein sequences. The protein sequences of *Arabidopsis thaliana *and *Oryza sativa *were obtained from the literature [6,8,11]; the protein sequences of *Populus trichocarpa *(angiosperm), *Selaginella moellendorfii *(fern), *Physcomitrella patens *(moss), and *Chlamydomonas reinhardtii *(green alga) were retrieved from JGI genome database (http://genome.jgi-psf.org) by tBLASTn search with default parameters (E value = 1e-5). To better understand the evolutionary history of plant class V SET genes in land plants, we also included Su(var)3-9 SET protein sequences from other plant species, including incompletely sequenced *Nicotiana tabacum *(angiosperm), *Vitis vinifera *(angiosperm), *Ricinus communis *(angiosperm) and *Pinus taeda *(gymnosperm), either by BLASTp from NCBI protein database (nr) or from TIGR EST databases [46]. The protein sequences of SET and PreSET domain regions from 7 Su(var)3-9 SET proteins in *Arabidopsis *were used as the queries. In the JGI database, if alternative splicing was present in the gene model, only the longest transcript was selected, and if truncated SET proteins were found, their gene models will be re-predicted using genomic scaffold sequences. Protein domains were predicted by SMART [47] and Pfam [48] platforms and the sequences possessing PreSET and SET domains are regarded as the candidate Su(var)3-9 SET proteins.

### Sequence alignment and phylogenetic analysis

We used the protein sequences of PreSET and SET regions to construct a combined dataset. Alignments of these two regions were first generated independently at the amino acid level using Clustal X [49], followed by manual adjustment, and then the combined matrix of protein sequences was constructed for 73 plant *Su(var)3-9 SET *genes. The corresponding codon alignment was also constructed according to the protein sequence alignment using the PAL2NAL program [50] for gene structure analyses. Phylogenetic analyses were performed using protein sequences. PHYML [51] and MEGA 3.1[52] were used for ML [53] and NJ [54] analyses, respectively. For the ML method, the ProTest [55] program was used for testing evolutionary model and optimizing parameters. For the NJ method, we used the Jones-Taylor-Thorton +Γmodel as well as simple models of amino acid replacement, such as p-distance [56] with pairwise deletion of gaps. Supports were estimated by non-parametric bootstrap using 1000 replicates for the NJ tree and 500 replicates for the ML tree. In this paper, we used the following descriptions and ranges in the text for describing bootstrap support: weak, 50-75%; moderate, 76-85%; strong, 86-100%.

### Analysis of gene structure

Gene structure was analyzed on the basis of phylogenetic analysis. Our analyses mainly focused on the PreSET and SET domain regions because the regions outside of these two domains are highly variable in plant Su(var)3-9 SET proteins. Intron-exon borders were determined by aligning the cDNA sequences to their respective genomic region with the spidey program [57] followed by manual inspection of the splice consensus signals. Intron phase was analyzed manually based on the intron-exon border information: phase 0 designated introns between codons, phase 1 designated introns between the first and second bases of a codon, and phase 2 designated introns between the second and third bases of a codon. The intron position information was obtained from nucleotide sequence alignments derived from the protein alignments. Intron positions that are apart even by one base pair were considered as non-identical even if it cannot be excluded that they might have the same ancestor [58].

## Authors' contributions

ZDC, HM and XYZ designed this study. XYZ carried out data searches and analyses, and drafted this manuscript. HM, and ZDC revised several versions with input from all the authors. All authors have read and approved the final manuscript.

## Supplementary Material

Additional file 1 **Plant *Su(var)3-9 SET *homologues and related genes surveyed**. The MS excel file provides sampling information of plant *Su(var)3-9 *SET homologues and related genes in ten plant species. Click here for file

Additional file 2 **73 protein sequences used in this study**. A txt file gives all protein sequences with fasta format used for phylogenetic analyses. Click here for file

Additional file 3 **Alignment of 73 protein sequences**. A txt file provides an alignment of 73 protein sequences with 228 sites. Click here for file

Additional file 4 **NJ tree with branch lengths**. A single NJ tree with branch length proportional to the amount of change. The numbers above branches are bootstrap percentage >50. JTT model was used. Click here for file

Additional file 5 **Alignment of 24 mRNA sequences with intron position information**. The string of dots indicates the alignment region not containing any intron position information. The red arrows denote the positions of introns. Click here for file

Additional file 6 **Genomic DNA sequences and corresponding mRNA sequences**. A zip file provides the 24 genomic DNA sequences and corresponding mRNA sequences used for gene structure analysis with fasta format containing a concise annotation. Click here for file
