# Supplementary figures and images for: Phylogenetics and evolution of Su(var)3-9 SET genes in land plants: rapid diversification in structure and function
Source: BMC Evol Biol. 2011 Mar 9;11:63. doi: 10.1186/1471-2148-11-63 (PMC3063831; doi:10.1186/1471-2148-11-63)

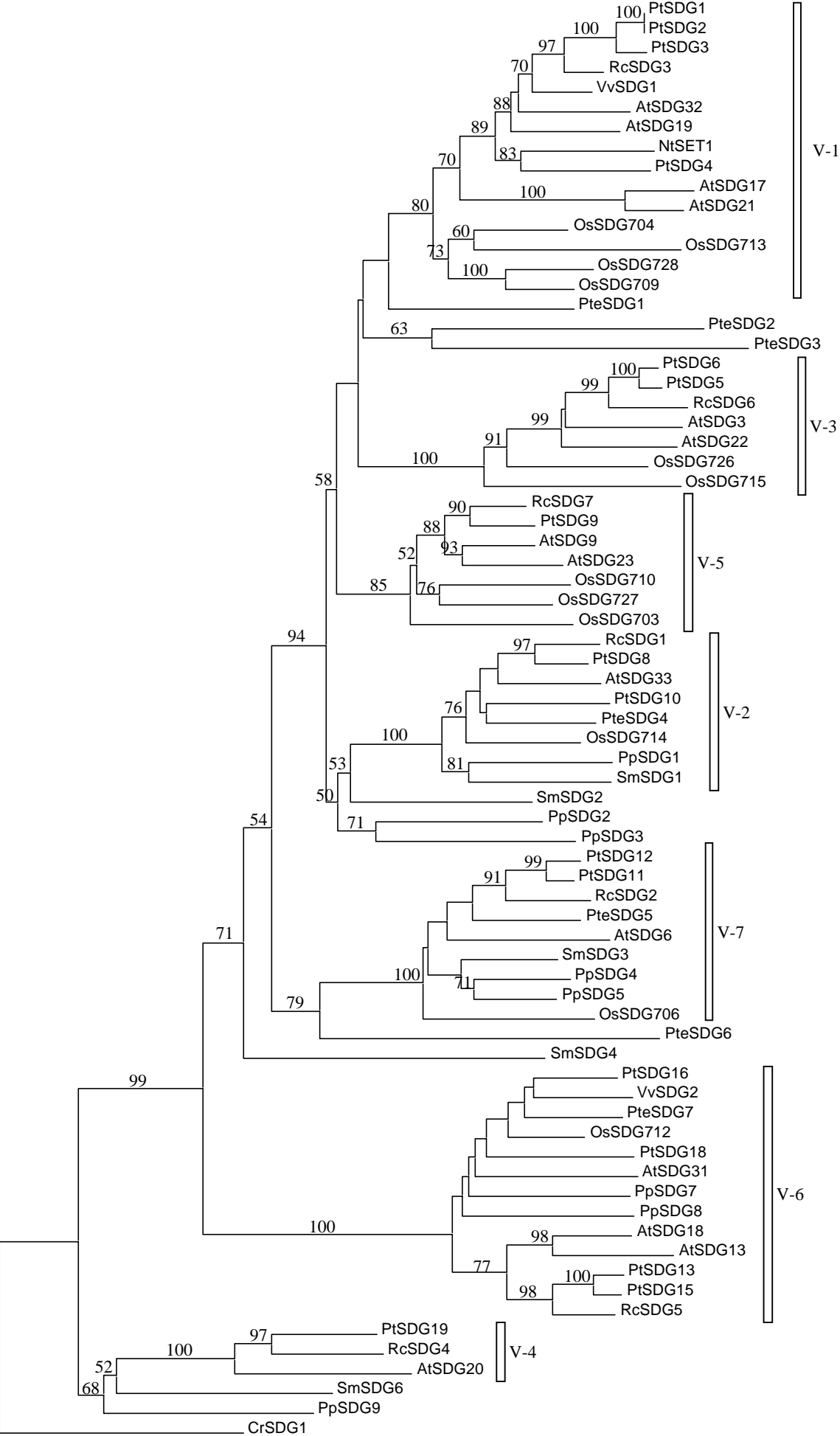

0.1

Supplement: Additional file 4 — NJ tree with branch lengths. A single NJ tree with branch length proportional to the amount of change. The numbers above branches are bootstrap percentage >50. JTT model was used. [file 1471-2148-11-63-S4.PDF]
