# Supplementary material for: Phylogenetics and evolution of Su(var)3-9 SET genes in land plants: rapid diversification in structure and function
Source: BMC Evol Biol. 2011 Mar 9;11:63. doi: 10.1186/1471-2148-11-63 (PMC3063831; doi:10.1186/1471-2148-11-63)
Supplement: Additional file 5 — Alignment of 24 mRNA sequences with intron position information. The string of dots indicates the alignment region not containing any intron position information. The red arrows denote the positions of introns. [file 1471-2148-11-63-S5.DOC]

#AtSDG33 AGT GAA ATT TGT GTC GAT GAC ACT GAG TTA CAT GAG AAA GAA GAG GAA AAT GTC GAT GGG AGC CCT AAA --- --- --- [ 78]

#PtSDG10 CAG AGC ACG AAG TTA GAT GCT CCT GAA CTC GAT ATC TGC AAG AAA GAA GTT GAA GGA GAG TCA ACA GGG CCT GGA GAG [ 78]

#OsSDG714 GAA GAG GAG AAG AAG GAG CCG GTG GCG CAG CAC GCC ATG AAG CCG GTC AGG ATG GGC GAT GCA GCC TCG --- --- --- [ 78]

#SmSDG1 ATG AGG CTC AAG ACT ACT GGA ATG AGC GCC GAT CTT GCC GAT CTT GCG AAG GTA GGG GCA GAG GGC --- --- --- --- [ 78]

#PpSDG1 --- --- --- --- --- --- --- --- --- --- --- --- --- --- --- --- --- --- --- --- --- --- --- --- --- --- [ 78]

#AtSDG13 GAA GAG GAT GCT TAT AAG GTC CTT CTT GAT GCA ATC TTC GAC GAG GCT GAT GCC CAG TCC ACT GAA AAA AAT AAG AAG [ 78]

#AtSDG18 GCT GAA GAC AAT TAC AGG GTT CTT GCT GAT GCT ATC TTC GAT AGC CAT GAA GAT CAG GCT ATT CAA GAA AGC --- --- [ 78]

#AtSDG31 AAG TTG GAT AAC TAT ACT GCA CTG GTC GAC GCT ATT TAT TCT GTT --- --- --- --- --- --- --- --- --- --- --- [ 78]

#PtSDG15 GAG GAG GAA AAT TAT AGA GCT CTT GCG GAT GCT ATC TTT GAA GAG GAG GAG GCT AAG GTT CCG GAA GAG AAG GAT GAG [ 78]

#PtSDG18 GAA GAA GAT AAT TAT AAA GAA CTG ATT GAT ATT CTA ACA AAG GAG --- --- --- --- --- --- --- --- --- --- --- [ 78]

#OsSDG712 GAA GCT GAT AAT TAT CTG GCT CTT GCT GAT GCT ATA TTT TGT GAT --- --- --- --- --- --- --- --- --- --- --- [ 78]

#PpSDG7 CTT GAA AAT CTC TAC CGG ATT TTG GGT GGA TTG CGT TCT GGC GTG GTG GGT --- --- --- --- --- --- --- --- --- [ 78]

#PpSDG8 GAT CGT GAC AAT CTC GAG GAG TAT CGG TCG ATG ATT CCC GTG ACA AGC GAA CCA CAA TGC TTA AAT CCT GAA --- --- [ 78]

#AtSDG6 AAG TGT ATG CTT CTT CAG TGT ATT CCT TGT GGC AGC CAT TTT GGA GAT AAA GAA CAG TTG CTG GTA CAT GTG CAA GCT [ 78]

#PtSDG11 CAG TGC ATG CTG TTC CAG TGC ATT CCT TGT GCC AGC CAC TTT GGG AAC ACT GAC CAG TTA TGG TTG CAT GTC CTC TCT [ 78]

#OsSDG706 --- --- --- --- --- --- --- --- --- --- --- --- --- --- --- --- --- --- --- --- --- --- --- --- --- --- [ 78]

#SmSDG3 CAC ATC TGT ATC AAC TGT GAC GAG CAG TTT CCA CAC TTC GAG GCT CTG TGG GAT CAC GTG GTG TCG GGA --- --- --- [ 78]

#PpSDG4 TCG CCG GCT TGG TCA GTG TGC GTG ATG TGT GAC AAA CAG TAT TTA GAT TTT GAT CGC CTA TGG CAG CAT GTG GAG GAT [ 78]

#PpSDG5 ATG ACT GTG TGC GTG ATA TGT GAC GAG AAG TTT AAG AAT TTT GAC CTG CTA TGG TTG CAT GTT GAG GAT CAA CAC TTC [ 78]

#AtSDG20 AAT --- TTG ATT CTC CCA TGG CTA AAC --- --- --- --- --- --- --- --- --- --- --- --- --- --- --- --- --- [ 78]

#SmSDG4 AAC GCC TTG AAA AAT CTT GGT AAA GCT CAG GAG TCC TAC TTG CGA GCC ATT CAA CTC CGG --- --- --- --- --- --- [ 78]

#SmSDG6 GAC GAA TGC CGC TAC CCT TAC TTC GAG --- --- --- --- --- --- --- --- --- --- --- --- --- --- --- --- --- [ 78]

#PpSDG9 GAC GCA GCT CAG GGA --- --- --- --- --- --- --- --- --- --- --- --- --- --- --- --- --- --- --- --- --- [ 78]

# Position 1

#CrSDG1 CAG GAA CAG CTG CCA GTG CCC GTC GAC CCG CCA CCA CAC ACC AGC --- --- --- --- --- --- --- --- --- --- --- [ 78]

．．．．．．．．．．．．．．．．．．．．．．．．．．．．．．．．．．．．．．．

#AtSDG33 --- --- --- CGA AGA AGC CCT --- --- --- --- --- --- --- CCA AAG CTA ACC GCA ATG CAG AAA GGA AAG CAG AAA [ 234]

#PtSDG10 CCT GAT AAG AGC AGG CCA TTA TAT GAT GGA GAT CAG AGG AGG GAG TTA GAT GCT CCT AAA CAT GAT GTG TGC AAG AAA [ 234]

#OsSDG714 --- --- --- GAG AGG AAG CCC TCC TCG GAG GGG AAG CCG ATG CCC GCA ATT GCG GCG GAG CCG GTT TCT TGT GCC GGG [ 234]

#SmSDG1 --- --- --- --- --- --- --- --- --- --- --- --- --- --- --- --- --- --- --- GAG GTG GAT GTG GAG AAT GGC [ 234]

#PpSDG1 --- --- --- --- --- --- --- --- --- --- --- --- --- --- --- --- --- --- --- --- --- --- --- --- --- --- [ 234]

#AtSDG13 --- --- --- --- --- --- --- --- --- --- --- --- --- --- --- GAA GAG GAA AAG AAG AAG AAG GAA GAG GAA AAG [ 234]

#AtSDG18 --- --- --- --- --- --- --- --- --- --- --- --- --- --- --- GAA GAA AAG AAG GCA GAC GAA GTT AAA GAG GAT [ 234]

#AtSDG31 --- --- --- --- --- --- --- --- --- --- --- --- --- --- --- GAG GAT GAG AAT AAG CAA AGT GAA GGT TCA TCT [ 234]

#PtSDG15 TTT GGT TTG CAG GAG GGG ACT TTG GAG GAA GAA ACT TTG GCA TCT AGT GAA CCT GAG TTG CCA TTA AAG CGG CTG CGA [ 234]

#PtSDG18 --- --- --- --- --- --- --- --- --- --- --- --- --- --- --- AGT AAT GAG GAA TCA AAC TTG AAT TAC AAT TTC [ 234]

#OsSDG712 --- --- --- --- --- --- --- --- --- --- --- --- --- --- --- TCA GAT CCC AAA GAA GGA CAG AAA AGG CAA GCT [ 234]

#PpSDG7 --- --- --- --- --- --- --- --- --- --- --- --- --- CTT TTT TGT TCT TCG CCA GCG GAA GCT GGT GGT ATT GTG [ 234]

#PpSDG8 --- --- --- --- --- --- --- --- --- --- --- --- --- --- CCC CTG CAG TCG GTA GTC CCA ACC TCG AAC TTA CTT [ 234]

#AtSDG6 CTA ACC GTT GCT TCT GAA TGC AAT TTG ACT AAT GGT --- --- --- GAG TTT TCT CAG AAA CCT GAA GCA GGC AGT TCA [ 234]

#PtSDG11 CCA AAA GGT GCT CAA CAA CTT AAT CCG TCT ATG GGT GAA GAG AAG GAG GAT TCC TTG CAA AAA CTT GAG CTG CAA AAT [ 234]

#OsSDG706 --- --- --- --- --- --- --- --- --- --- --- --- --- --- --- --- --- --- AAA AGA ACA GAA AGG GCA AGT GAT [ 234]

#SmSDG3 --- --- --- --- --- --- --- --- --- --- --- --- --- --- --- --- CAT TCG GAT CAT CTC AGT GCA ATG CCA ACA [ 234]

#PpSDG4 TTT GCA GGA CGA GTG AAG GCC TCA CTG ATG TTA CGC AAG GGG CAT AAA TGT ATT TTT TGC AGC GAA ACT TTT AGT ACA [ 234]

#PpSDG5 GTG TGT TGG GAA GAG TTT GAT ATA GAG TTG GAG GTT TGT AAT CAT AAA GGG ATT GTT CAC AAT GGC CTT TCG TCT TCA [ 234]

#AtSDG20 --- --- --- --- --- --- --- --- --- --- --- --- --- --- --- CCT CGA GAA CTC GCC GTT GTT GCT CAA ACC TGC [ 234]

# Position 2

#SmSDG4 --- --- --- --- --- --- --- --- --- --- --- --- --- CCA GAC TAT GCA ATT GCG TAC GAA AAT GCG CAA AAT ACT [ 234]

#SmSDG6 --- --- --- --- --- --- --- --- --- --- --- --- --- --- --- TAC TCG CCG GTG TCT GTT CTT GCG AGG GAG AGG [ 234]

#PpSDG9 --- --- --- --- --- --- --- --- --- --- --- --- --- --- CTG GAG CGG TGG CCT GTG CCG GTA CGC AAT GAC CTT [ 234]

#CrSDG1 --- --- --- --- --- --- --- --- --- --- --- --- --- --- --- CGA ACT GCG CCC CTG CCC CTG GAC ACC AGA TAC [ 234]

．．．．．．．．．．．．．．．．．．．．．．．．．．．．．．．．．．．．．．．

#AtSDG33 AGT ACT TCA GAA ATT GAG GGT TTG GTA TGT GAG GAC ATC TCC GGA GGG CTA GAA TTT AAG GGT ATC CCC GCC ACT AAT [1716]

#PtSDG10 ACT ATT GGC AAA TTG CAT GGC TTG GTG TGT GAG GAC ATC TCT TTT GGG AAA GAA GAC ATA CCT ATT CCT GTT ATC AAT [1716]

#OsSDG714 ACG ATT TCG GAA TTA CCT GGG TTG GTT TGT GAT GAC ATA TCT GGT GGG CAG GAG AAT CTT CCT ATT CCT GCT ACT AAT [1716]

#SmSDG1 GCG CCA TCT GAG --- CGC GGA CTG GTT TGC AAA GAT ATT TCC AAT GGA CTG GAA GTT TTG CCA GTT CCC GTG TCG AAT [1716]

#PpSDG1 AAC GTT TCA GAA CTG CGA GGA TTG GTG TGT AAA GAC ATC TCG AAT GGG CAG GAG CGC ATA CCA GTG CCC GCT TCT AAC [1716]

#AtSDG13 GGA GAT GGC TGG AAA GCA ATT AGC AAC ATG AAG GAC ATT ACG GCT GGT GAA GAA AAC GTT GAG ATT CCA TGG GTG AAT [1716]

#AtSDG18 GCA GAT GAG TGG AGA TTG ATA AGC AGC GTC GGG GAC ATC AGT CTA GGG AAA GAA ACT GTT GAG ATT CCC TGG GTG AAT [1716]

#AtSDG31 --- --- --- --- --- --- --- --- AGA ATT GCT GAC ATA ACT AAA GGT TCA GAG AGC GTT AAA ATC CCT CTT GTC GAT [1716]

#PtSDG15 GCT GAT GAG TTT AGG TTC CTT AAT TAC CAT TCT GAC ATT ACC AAG GGA GAA GAA ATG TTT GAG ATT CCA TGG TCG AAT [1716]

#PtSDG18 GTC CTT CAA AAG AAA TAC CCC ATT TAT CTC AAA GAT ATA TCT CGT GGT GAA GAT AAT ATA CCA GTT CCC CTG GTG AAT [1716]

#OsSDG712 ATT GGT GCC AAC AGA CCA CCT CAT GAT GTC AAT GAT ATC ACA AAA GGT GAA GAA CGT TTA AGG ATT CCA ATT ATT AAT [1716]

#PpSDG7 TAT GTG AAA GGC CAA TGT AGA CAT GAT GAC AAA GAC CTA TCT CGT GGG TTT GAA GCT ATT CCC ATC CCC ATT GTG AAT [1716]

#PpSDG8 GCA AGG AAG CCC GAC CAT CGC CAC GAT CGT TAC GAT ATC TCC CGA GGG AAG GAA AGA GTC CCC ATC TCG CTT AGC GCT [1716]

#AtSDG6 AGA CCG TTT GGA AAC ACT GCG GTT TTG TGC AAG GAC ATC AGC TTT GGT AAG GAA TCA GTT CCA --- ATT TGT GTC GTC [1716]

#PtSDG11 GGG CTT AAG CAA AAG GCT ACT GTC TTG TGT ACT GAC ATA AGC TTT GGG AAG GAG ACA ATA CCA GTG GCA TGT GTT GTA [1716]

#OsSDG706 AAT CTT AAG AAT GAG ATG GTT ATT GTT TGT GAA GAT GTT AGC TTT GGC AGG GAG AAA GTT CCA GTT GTC TGT GCC ATT [1716]

#SmSDG3 TCG GCC AGG AAA AAG AGC GTG GTT CTT TGC GAA GAC ATA AGC TTC GGG AAA GAG CGT GTA CCG GTT CCT TGT GTG GAG [1716]

#PpSDG4 AAT TCT ATC TCA AAC AAA ATG GTT CTA TCT GAA GAC TTG AGC AAT GGA TTG GAG AAA GTA CCT ATT CGT TGT GTC GTA [1716]

#PpSDG5 GCA GAT GCC TTT ATT TGT CCC AAT CAA TGT TCA CCC TTC CAT GCT GCG GGT GCA GCT GCT CCA CTT TTG GAT GTT GAC [1716]

#AtSDG20 --- --- --- --- --- --- --- --- --- --- --- GAC GCT GCA CGA TCC TTG GAA AAC ATC TCA --- --- --- --- ATC [1716]

# Position 4

#SmSDG4 AGG TTG AGG CAC ATA ACA ATG TTT GCC GCG AAA GAT ATT GCA GCC AGC GAA GAG TTG ACA TTT GAC TAC TCT GGT GCT [1716]

#SmSDG6 --- --- --- TTT GAT TAC GGG CAG TTT GGA GGC GAT GGT TGC CGC TGC ATT GAC TGC TGC CGA GGA GAA CAA GAA GAT [1716]

# Position 3

#PpSDG9 --- --- --- --- --- --- --- TCT TGT TGC CGC AAT GCA TCG AGG TGT GCT GAC CGT TGC GTT CTT GCA CAG CAG AAT [1716]

#CrSDG1 --- --- GAG GGT AAA GCG CAC GCA TCT GCG ACT GAC GCA GCA GCT CCC GCC GCC AAG CGC CCG CGC CGA TGC AAC AAT [1716]

#AtSDG33 CGT GTT GAT GAT TCA CCA GTT TCA CCA ACA TCT --- --- --- --- --- --- --- --- --- --- --- --- --- --- --- [1794]

#PtSDG10 GTA ATT GAT AAT CCT CCT ATA GCA CCT CCA GGC AAG TTA CTG ACC --- --- --- --- AAC TTA TGC TTT TAT GAG GGA [1794]

#OsSDG714 TTG GTT GAT GAC CCA CCT GTT CCT CCA ACT --- --- --- --- --- --- --- --- --- --- --- --- --- --- --- --- [1794]

#SmSDG1 CTC GTG GAC AAT CCG CCT TGT GCA CCC GAT --- --- --- --- --- --- --- --- --- --- --- --- --- --- --- --- [1794]

#PpSDG1 ACA GTC GAT GAT CCT CCA GTT CCT CCT ACA --- --- --- --- --- --- --- --- --- --- --- --- --- --- --- --- [1794]

#AtSDG13 GAG ATC --- AAC GAG AAG GTT CCT TCC CGT TTC CGT TAC ATG CCT --- --- --- --- --- --- --- --- --- --- --- [1794]

#AtSDG18 GAG GTC --- AAC GAC AAA GTT CCT CCA GTT TTC CAT TAC ATA GCC --- --- --- --- --- --- --- --- --- --- --- [1794]

#AtSDG31 GAT GTT GGG AGT GAA GCT GTG CCA --- AAG TTT ACT TAC ATC CCT --- --- --- --- --- --- --- --- --- --- --- [1794]

#PtSDG15 GAA GTA --- AAC AGC GAG TTT CCC CCA GTT TTT AAC TAC ATA CCC --- --- --- --- --- --- --- --- --- --- --- [1794]

#PtSDG18 GAA AGC --- AGC ACA CTA GAG TTG CCA GAT TTT ATC TAC ATA AAA --- --- --- --- --- --- --- --- --- --- --- [1794]

#OsSDG712 GAA TAT GGC AAT GGG ATT CTT CCT CCT CCA TTT CAC TAC ATA CCA --- --- --- --- --- --- --- --- --- --- --- [1794]

#PpSDG7 CAT ATT AAC TCG GAG ACT CTC CCT TCC TCC TTC TTC TAC ATT GAT --- --- --- --- --- --- --- --- --- --- --- [1794]

#PpSDG8 CTC GGT GCG GAG GAC TTG CCG GAG GAA TTC TTC TAC ACG AAG AGC --- --- --- --- --- --- --- --- --- --- --- [1794]

#AtSDG6 GAT GAT GAT CTT TGG AAT TCT GAA --- --- --- --- --- --- --- --- --- --- --- --- --- AAA CCG TAT GAG ATG [1794]

#PtSDG11 GAT GAA GAT CTC ATG GAT TCC CTC CAT GTC TTG GCA GAT GGT TAT --- --- GAT GGT CAA ATT AGT AAA TTC CCC AAG [1794]

#OsSDG706 GAT GTA GAT GCA AAA GAA TTT CCA TAT ATG AAA CCT GGA --- --- --- --- GAA ATT TTG CAA AGT GAA AAT TCT CTG [1794]

#SmSDG3 GAC GAA GAG ACC GAT CCC TGC AAC TGC TTG CAG TGT AGG GAG GGA --- --- AAG TCG TAC CAC GTC GAC TCT CTA AAA [1794]

#PpSDG4 GAT GGA AGT GTG ATA GAG CCT TGC ACG TGT TCG TTG TGT ACG GAG GGT GGA AGC CTA ACA TCT TCA GGG GAC AGT CAA [1794]

#PpSDG5 ATG GCT GGA TTT TCT GAA GCC CCT TTC AAA GCC CAA GCT GGG CCA --- --- --- --- --- --- --- ACA CTT CTG AAG [1794]

#AtSDG20 CCG TTT CAC AAC TCC ATC GAT TCT CAA CGA TAC GCG TAC TTC ATC --- --- --- --- --- --- --- --- --- --- --- [1794]

#SmSDG4 CAA CAA CTT AAA TCT CCA ACG ACA ACC TCA CCT GAT ACT CAT CTT CTG CAC --- --- --- --- --- --- --- ATG AAA [1794]

# Position 5

#SmSDG6 CCA GGA TTT CCT TCA CAG GCA CCG GAT GAT TTG ACT AGC GCG ATC --- --- --- --- --- --- --- --- --- --- --- [1794]

#PpSDG9 CAT GGC GAT GGT AAA CAT GTC ATA TCT GAC GAT ACG AAT TCT ACG --- --- --- --- --- --- --- --- --- --- --- [1794]

#CrSDG1 GAC GAC AGC AGT AGC AGC CGT GGC GCA ACC GGC AGG CGT AGT TGC AGT --- --- --- --- --- --- --- --- --- --- [1794]

# Position 6

#AtSDG33 --- --- --- GGT TTC ACA TAC ATC AAA TCT TTG ATT ATT GAG CCT AAT GTC ATA ATT CCA AAG AGT TCA ACT --- --- [1872]

#PtSDG10 AGT GGA TCT GGC TTT AAA TAT ATC AAG TCT GTT CAA GTT GCA AGA AAT GTG ATC ATT CCT CCA AGT GCC TCT --- --- [1872]

#OsSDG714 --- --- --- GGT TTT GTG TAC TCC AAA TCT CTA AAA ATT CCA AAG GGC ATC AAG ATT CCA TCT TAT TGT AAT --- --- [1872]

#SmSDG1 --- --- --- GGC TAC AGA TAC ATC AAC AAA ATC GAA ATT GAT GAC GGG ATT GTA CTT CCG CCT CCA GCG CTG --- --- [1872]

#PpSDG1 --- --- --- GAT TAC ACG TAC ATC ACG AAG ACC GTA GTA CCA GAC GAC ATT GCA AGA CCT CCC CCT TCT AAA --- --- [1872]

#AtSDG13 --- --- CAC AGC TTT GTG TTT CAA GAT GCT CCA GTG ATA TTC TCG CTC TCA AGT TTC TCT GAT GAG CAA AGC TGC TCC [1872]

#AtSDG18 --- --- CAA AGT CTT GTG TAT CAA GAT GCT GCA GTG AAG TTT TCG CTT GGG AAT ATC AGG GAT GAC CAA TGC TGC TCC [1872]

#AtSDG31 --- --- CAC AAC ATT GTT TAC CAA AGT GCT TAT CTC CAC GTG TCT CTG GCT CGA ATC TCT GAT GAA GAT TGC TGC GCA [1872]

#PtSDG15 --- --- CGA AAT TTG ATT TTT CAA AAT GCA TAT GTC AAC TTC TCT CTC TCA CAA ATT AGG GCT GAA AAT TGT TGC TCG [1872]

#PtSDG18 --- --- AAT AAC ATG GTA TAC CAA GGT GGC CAT GTT GAT TTT TCT CTT GCT CGA ATA TCA GAA GAT AAC TGT TGT GCA [1872]

#OsSDG712 --- --- CAC AAT ATC ACA CTC CAA GAA GCC TAT GTA AAC ATC TCC CTT GCT AGA ATC GGA GAT GAC AAT TGC TGT TCT [1872]

#PpSDG7 --- --- AAG AGC AGA CCA TAT GAA AAA GCT TTC GTA AAC CTT GCT ATC TCG CGA ATA GGA GAC GAC GAT TGC TGT CCA [1872]

# Position 8

#PpSDG8 --- --- --- AGC GTG GTG TTC CAA AGT GCG CAT GTG GGT ATA TCC ATG GCG AGG ATC GGG GAG GAC GAT AGG TGC AGT [1872]

#AtSDG6 CCT TGG GAA TGC TTT ACT TAT GTT ACA AAC TCA ATT CTT CAT CCG TCA ATG GAT CTT GTA AAG GAG AAT CTG CAA CTC [1872]

#PtSDG11 CCC TGG GAT ACT TTT ACC TAT GTA ACA GGG CCA GTT CAT GAT CAA TGT GAT AGT CTG GAC ATT GAG GGT TTG CAA TTG [1872]

#OsSDG706 CCC TGG CAA GGT TTC CAC TAC GTC ACA AAG CGT TTG ATG GAT TCA TCT CTT --- GTT GAT TCA GAG AAC ACT ATG GTT [1872]

#SmSDG3 CCG TGG GAG ACT TTC AGC TAC GTC ACA GAT CGT TTG CTT GAT CCG TCT TTG GGA CTT GAT ACG AAG GAT TCG CAA ATC [1872]

#PpSDG4 CCC TGG AAT AAC TTT GTA TAT ATA ACT CAA CGG CAC CTT GAC CCT TCA TTG GGT CTA GAT ACG AAG AGC TCT CAA GTA [1872]

#PpSDG5 ACT ATC GAT ATG AAA GAT TTT ATG AAA GGA AAG CAT ATG GTG ATC CAT GAG GAT TTG AGT AAT GGC CAA GAA CCA GTA [1872]

#AtSDG20 --- --- TAC ACA CCT TTT CAG ATC CCC GCT TCA TCT CCT CCT CCG CCG CGA CAA TGG TGG GGA GCT GCT GCT AAT GAA [1872]

#SmSDG4 TAC GAA GAA TTA GCA GCA GAA GGC ACA GGC ACA GCA CCT CTA CAC CCA TTT CCT ACA GCA TTC CAC AGC GGG ATT TGT [1872]

# Position 7

#SmSDG6 --- --- GAA CTT TTG ACT TTG ATC CAG GAT GAA GAT GAT GAT AGC GAC GAA GCA CTC CCG AGC AAA AAG AAA AGG CGG [1872]

#PpSDG9 --- --- TGT GCC TGC GGG TTC ACA AGC GAC GGA GTG AAC GCA TAC ACG CAC GAG TCC AAG ATG CAG TTA GTG GAC AAA [1872]

#CrSDG1 GGC AGT AGC GGC AGT AGC GAG GAG AGC GAT GGA GAG GGG TGG AGC TGC TGC GGG TGT GGC GTG CTC ATG CCG GCG GGT [1872]

#AtSDG33 GGG TGT AAC TGC CGA GGC AGC TGC ACT GAC TCA AAG AAA TGT GCA TGT GCT AAG CTT AAT GGG --- --- --- --- --- [1950]

#PtSDG10 GGG TGT GAT TGT AAA GGA AAA TGT ACA AAT CCA AGG TCT TGT TCT TGT GCT CGA CTT AAT GGC --- --- --- --- --- [1950]

#OsSDG714 GGC TGT GAC TGT GAA GGA GAT TGC GCA AAC AAC AAA AAC TGC TCA TGC GCG CAG CGC AAT GGT --- --- --- --- --- [1950]

#SmSDG1 GGA TGC AGT TGC AAG GGG CTC TGC GTT GAT CCT AAA ACA TGC AGC TGT GCA AAA CGA AAT GGA --- --- --- --- --- [1950]

#PpSDG1 GGC TGC AGT TGC AGG GGC GCC TGT ACA GAA GAG AAG GAC TGT GCT TGT GCT CGC AAG AAT GGA --- --- --- --- --- [1950]

#AtSDG13 ACT TCT TGT ATC GAA GAC TGC CTG GCT TCA GAA ATG TCG TGC AAT TGT GCA ATT GGT GTT GAT AAC GGG TTT GCG TAC [1950]

#AtSDG18 --- TCT TGC TGT GGA GAT TGC TTG GCT CCG TCA ATG GCA TGC AGA TGT GCA ACT GCT TTT AAT --- GGC TTT GCA TAC [1950]

#AtSDG31 --- AAC TGC AAA GGG AAC TGT CTT TCA GCT GAC TTT CCT TGC ACT TGT GCT CGT GAA ACC AGT GGA GAA TAT GCT TAT [1950]

#PtSDG15 --- GCT TGT ATT GGT AAT TGT TTG TCG TCA TCT ACG CCT TGT GTT TGC TCC AGC GAC AGT GAG CAT GGT TTT GCA TAT [1950]

#PtSDG18 --- CAA TGT CTT GGA GAT TGT CTA TCC TCA GAC TTG CCT TGT GCA TGT GCT GCG GAA ACA GGT GGC GAG TTT GTT TAC [1950]

#OsSDG712 --- GAT TGT TTC AGA GAT TGT CTG GCA CAA TCA CTT CCT TGT GCG TGT GCT GCA GAA ACA GGA GGA GAG TTT GCT TAT [1950]

#PpSDG7 --- AAT TGC CAC AAT GAT TGC CTA TCT GCC CCC TAT CTC TGC GCT TGT GCC CGG GAG ACA GGT GGA GAG TTT GCA TAT [1950]

#PpSDG8 --- GGC TGT GTG GGC AAC TGT CTC GAC AAG CTC ACT CCC TGC GAG TGC GCG AGG CTG ACT GAC GGA GAG TTC GCG TAC [1950]

#AtSDG6 AGG TGT AGC TGT CGC AGT TCA GTG TGC TCG CCT GTA ACT TGT GAT CAC GTA TAC CTT TTC GGT AAC GAT TTT GAG GAC [1950]

#PtSDG11 AGG TGT TCT TGC CAA TAT TCA ATG TGC TGT CCT GAA ACA TGT GAT CAT GTT TAT CTC TTT GAT AAC GAC TAT GAA GAT [1950]

#OsSDG706 GGG TGT GCT TGT TCT CAT GCC CAT TGC AGT CCT GAG GAA TGT GAT CAT GTG AGC CTC TTT GAT AGT ATC TAT GAG AAC [1950]

#SmSDG3 GGC TGT TCT TGC GGA CGG GGT CGA TGC TCA TCA AGG TCG TGT GAT CAC GTG GAA ATG TTT GAT AGT GAT TAC GCA GAC [1950]

#PpSDG4 GGA TGT TCA TGC ACT GGA GAT GAA TGC TCG GCG TCC ACT TGT GAT CAT GTG TCG ATG TTT GAC ACT GAC AAC GCT GAA [1950]

#PpSDG5 CCA ATT CCA TGT GTG ATT GAT GAA GAT CTT TTA AGG CCC TGT ACA TGT GCC AAT TGT TGT GAA AAT GGC ATA AAC GCT [1950]

#AtSDG20 --- --- --- --- --- --- TGT GGA TCG GAG TCT AGA CCT TGT TTT GAC TCA GTG AGT GAA AGC GGA CGT TTT GGG GTG [1950]

#SmSDG4 AGG CCG ACC ATC ATG CAG GAA AAC AAA TCA GAA TGC TCT TGC TTG CGA ATG CGG CTC CGT GAT GAT TCT GGT TTG TTC [1950]

#SmSDG6 --- --- --- --- --- --- CTC TCG TCC AGC TCC TGT CCC TGC GGT CGG ACG ATC TTC GGC GAG CGA GCA TAC GAT TCA [1950]

#PpSDG9 CGT AGC AAT GGG ACA GAT GCG TCT AAA AAT AGG ATT GAC TCA AAG TCC ATC TTT TCT AAT GAG GTT TCA TTG AAG GCC [1950]

# Position 9

#CrSDG1 GTC CGC GCC TAC CAG CCG AAC GGG AGG GGC CTG AAG CCC GAG CTG CTT GCA CAG GCG TCC GCT GCT GCC TTC ATC CTG [1950]

#AtSDG33 --- --- --- --- --- --- --- --- --- --- GGT AAC TTT CCA TAT GTT GAC CTT AAT GAT --- --- --- --- --- --- [2028]

#PtSDG10 --- --- --- --- --- --- --- --- --- --- TTT GAC TTT CCA TAC GTG GAC ATA GAT GGT --- --- --- --- --- --- [2028]

#OsSDG714 --- --- --- --- --- --- --- --- --- --- TCT GAT TTA CCT TAT GTA TCA CAC AAG AAC ATT --- --- --- --- --- [2028]

#SmSDG1 --- --- --- --- --- --- --- --- --- --- CAC ACG TTT CCA TAC GTT GAC AGC CAT GGT --- --- --- --- --- --- [2028]

#PpSDG1 --- --- --- --- --- --- --- --- --- --- ATG TCC TTC CCT TAT GTT TTC AAT CAT GGC --- --- --- --- --- --- [2028]

#AtSDG13 ACA TTA GAT GGG TTA CTC AAA GAA GAA TTC TTG GAA GCT CGG ATC TCT GAG GCT CGT GAT CAA CGG AAA CAG GTG CTG [2028]

#AtSDG18 ACA GTA GAT GGC CTC CTA CAG GAA GAT TTT CTG GAA CAG TGT ATC TCT GAG GCC CGT GAT CCG CGG AAA CAA ATG TTA [2028]

#AtSDG31 ACC AAA GAA GGG CTG CTA AAG GAA AAG TTT CTG GAC ACC TGT CTT AAG ATG AAA AAG GAA CCA GAT TCG TTC CCT AAA [2028]

#PtSDG15 ACT TTA GAA GGC CTT GTC AAA GAG GAC TTC TTA GAA GAT TGT ATC TCT CTA ACT CGG AAT CCT CAA AGA CAA TTT CTT [2028]

#PtSDG18 ACG CAG AAG GGA ATG CTG AAG GAA GAA TTT TTG GAT GAA GCC ATT GCC GTG TCT CTG GAT CCT CAA AGA AAA CAC TTC [2028]

#OsSDG712 ACA ACA GAT GGC CTT CTT AAG GGA GCA TTT CTA GAT AGC TGT ATC TCA ATG ATT CGA GAA CCA CTT AAA CAT CCC CAT [2028]

# Position 10

#PpSDG7 ACT TCA GAT GGC TGC TTA CAT CGA CGT TAC ATC GAT CAG CGG CAC AAG AAT GAA GAG AAC CCG --- --- --- --- --- [2028]

#PpSDG8 ACA GTG GAA GGG CTT CTG TAT CCC CAC TTT CTG AAG CAA GAG CTG GAT CGG AAG AGG AAC TTG AGC TTT CTG TCG TTC [2028]

#AtSDG6 GCT AGA GAC ATA TAT GGA AAA TCC ATG AGG TGT AGA TTC CCA TAT GAT GGC AAA CAA CGT --- --- --- --- --- --- [2028]

#PtSDG11 GCA AAG GAC ATA TAT GGG AAA TCC ATG CTT GGC AGA TTT CCG TAT GAC TAT AAA GGG CGG --- --- --- --- --- --- [2028]

#OsSDG706 CTA GTG GAT CTC CAT GGT GTA CCT ATG CGT GGT AGA TTT GCA TAT GAT GAA AAT AGC AAA --- --- --- --- --- --- [2028]

#SmSDG3 GCA TGT GAC ATA TGG GGA GAA GAA ATG CTT CGG AGG TTT CCT TAC GAT GGA GAA GGC CGC --- --- --- --- --- --- [2028]

#PpSDG4 GCC CGC ACA ATT GAT GGA AAG TCC GCT CGA GGC CAA TTT CCT TAT GAT GAA ATT GGC CGT --- --- --- --- --- --- [2028]

#PpSDG5 GCT CTG GAG GTT GCC GAG CCG TGG AAA ACA TTC AGC TAC ATC AAT AAA AGG CTG CTG GAC CCG TCA CTA GGT CTC GAT [2028]

#AtSDG20 AGT TTA GTG GAC GAG TCA GGA TGC GAA TGC GAG AGG TGT GAG GAA GGA TAC --- --- --- --- --- --- --- --- --- [2028]

#SmSDG4 GGG GAC CAA GAT TTA AGG TGT GAA CAT TTT CTT GGG TTG ACA GTG AAA TCG TTG TCT TGG AAT --- --- --- --- --- [2028]

#SmSDG6 AAC TCT CCC AGG CTC TTG AGT TTC CAA AAC CAG AGA GAA GAA GAA --- --- --- --- --- --- --- --- --- --- --- [2028]

#PpSDG9 AAT GAT GCC TCA GAA AAT ATG ACT TTC TGG ACT GAG TGG AAG TCC CTC GAA CTT TCT GAT GAC --- --- --- --- --- [2028]

#CrSDG1 GAA TGT GGC CCC GCA TGC GCA TGT CGC CAC CCG GCC CAG CCC GCG CAC AGC ACC CGC GGC --- --- --- --- --- --- [2028]

#AtSDG33 --- --- --- --- --- --- --- --- --- --- --- --- --- --- --- --- --- --- --- --- --- --- --- --- --- --- [2106]

#PtSDG10 --- --- --- --- --- --- --- --- --- --- --- --- --- --- --- --- --- --- --- --- --- --- --- --- --- --- [2106]

#OsSDG714 --- --- --- --- --- --- --- --- --- --- --- --- --- --- --- --- --- --- --- --- --- --- --- --- --- --- [2106]

#SmSDG1 --- --- --- --- --- --- --- --- --- --- --- --- --- --- --- --- --- --- --- --- --- --- --- --- --- --- [2106]

#PpSDG1 --- --- --- --- --- --- --- --- --- --- --- --- --- --- --- --- --- --- --- --- --- --- --- --- --- --- [2106]

#AtSDG13 CGA TTC TGT GAA GAG TGC CCA --- --- --- --- --- TTG GAG AGA GCT AAA AAG GTT --- --- --- --- GAG ATT TTG [2106]

#AtSDG18 CTG TAT TGC AAA GAA TGT CCT --- --- --- --- --- TTA GAG AAA GCC AAA AAA GAA --- --- --- --- GTA ATC CTG [2106]

#AtSDG31 GTT TAC TGC AAA GAC TGC CCT --- --- --- --- --- TTG GAG AGA GAT CAC GAT AAG GGC --- --- --- --- ACA TAT [2106]

# Position 12

#PtSDG15 TTT TAC TGT AGA GAT TGC CCA --- --- --- --- --- CTG GAA AGA TCA AAA AAT GAT --- --- --- --- GAG ATG TTG [2106]

#PtSDG18 TAC TAC TGT GAA ATC TGC CCC --- --- --- --- --- CTG CAG AAT GAA CCG CAA CAG AGA TAT --- --- GGG AAA ATA [2106]

#OsSDG712 TTC TAC TGC AAG ATT TGC CCA AAC GAA CGA ATG AAG ATA GAA GTA AAT TCT GAT TCA TCA AAC ACA GAA ATG AAT CCT [2106]

#PpSDG7 --- --- --- --- --- --- --- --- --- --- --- --- --- --- --- --- --- --- --- --- --- --- --- --- --- --- [2106]

#PpSDG8 TGC CTG CCA GGG ACT TGT CCT GTC GAG AGA --- --- --- --- --- --- --- --- --- --- --- --- --- ACG GGC GAC [2106]

#AtSDG6 --- --- --- --- --- --- --- --- --- --- --- --- --- --- --- --- --- --- --- --- --- --- --- --- --- --- [2106]

#PtSDG11 --- --- --- --- --- --- --- --- --- --- --- --- --- --- --- --- --- --- --- --- --- --- --- --- --- --- [2106]

#OsSDG706 --- --- --- --- --- --- --- --- --- --- --- --- --- --- --- --- --- --- --- --- --- --- --- --- --- --- [2106]

#SmSDG3 --- --- --- --- --- --- --- --- --- --- --- --- --- --- --- --- --- --- --- --- --- --- --- --- --- --- [2106]

#PpSDG4 --- --- --- --- --- --- --- --- --- --- --- --- --- --- --- --- --- --- --- --- --- --- --- --- --- --- [2106]

#PpSDG5 ACT GAG AGT TCG AAA CTT GGA --- --- --- --- --- --- --- --- --- --- --- --- --- --- --- --- --- --- --- [2106]

# Position 11

#AtSDG20 --- --- --- --- --- --- --- --- --- --- --- --- --- --- --- --- --- --- --- --- --- --- --- --- --- TGC [2106]

#SmSDG4 --- --- --- --- --- --- --- --- --- --- --- --- --- --- --- --- --- --- --- --- --- --- --- --- --- TCA [2106]

#SmSDG6 --- --- --- --- --- --- --- --- --- --- --- --- --- --- --- --- --- --- --- --- --- --- --- --- --- --- [2106]

#PpSDG9 --- --- --- --- --- --- --- --- --- --- --- --- --- --- --- --- --- --- --- --- --- --- --- --- --- --- [2106]

#CrSDG1 --- --- --- --- --- --- --- --- --- --- --- --- --- --- --- --- --- --- --- --- --- --- --- --- --- --- [2106]

# Position 13

#AtSDG33 --- --- GGC AGA TTA ATT GAG TCT CGA GAT GTT GTA TTT GAA TGT GGT CCT CAC TGT GGG TGT GGG CCA AAA TGT GTC [2184]

#PtSDG10 --- --- GGC AGA TTG ATT GAA GCA AAG GAT GTG GTG TTT GAA TGT GGT CCT GGA TGT AGC TGT GGA CCT AGT TGC ATT [2184]

#OsSDG714 --- --- GGC AGG TTG GTG GAG CCC AAA GCT ATT GTA TTT GAA TGT GGT GCT AAT TGC AGC TGC AAC AAC AAC TGT GTA [2184]

#SmSDG1 --- --- GGC AGA CTG GCT GTG CCT CTT GAC GCC GTG TAT GAG TGT GGC CCA AAC TGC GGG TGC GGT CCG GCA TGT ATA [2184]

#PpSDG1 --- --- GGA AGG CTG GTG AAA CCC ATG GAT GTT GTT TTT GAG TGT GGC CCT GGG TGT GGT TGT GGC CCT GAA TGC TTG [2184]

#AtSDG13 GAA CCA TGT AAA GGG CAT CTA AAG AGG GGA GCC ATT AAA GAG TGT TGG TTC AAA TGT GGT TGC ACT AAG AGA TGC GGT [2184]

#AtSDG18 GAA CCG TGC AAA GGG CAT CTA AAG AGA AAG GCC ATT AAA GAA TGT TGG AGC AAG TGT GGC TGC ATG AAG AAT TGT GGC [2184]

#AtSDG31 GGA AAA TGT GAT GGA CAC TTA ATC CGA AAG TTC ATC AAG GAA TGC TGG AGA AAG TGT GGA TGT GAT ATG CAG TGT GGA [2184]

#PtSDG15 GAA CCA TGC AAG GGT CAC TTG AAG AGG AAA TAT ATT AAA GAA TGT TGG AGC AAA TGT GGC TGC CAC AAA CAG TGT GGA [2184]

#PtSDG18 AAA CGA TGC AAG GGC CAT TTA ACT AGG AAA TTT ATA AAA GAG TGC TGG AGT AAA TGT GGA TGC AAC AAG AAA TGT GGA [2184]

#OsSDG712 GGT CCT TGT AAA GGA CAC CTT ACA AGG AAA TTC ATC AAG GAG TGC TGG AGA AAA TGC GGC TGC ACT AGA AAT TGT GGA [2184]

#PpSDG7 ACG AGT TGT AAA GGA CAT CCT GTG CGA GAC TTC TTG AAG GAA TGT TCA TCC AAG TGT GGA TGC AGC AAA CAA TGT GGC [2184]

#PpSDG8 GAG CCT TGC AAA GGC CAC ACT CAA CGA CGC TTC ATC AAG GAG TGT TGG GAG AAA TGT GGG TGC AAA CAG CTT TGC GGG [2184]

# Position 14

#AtSDG6 --- --- --- ATC ATC TTG GAG GAG GGT TAT CCT GTT TAT GAA TGC AAT AAG TTC TGT GGA TGC TCT AGA ACA TGT CAG [2184]

#PtSDG11 --- --- --- CTT GTT CTG GAG GAA GGT TAC CTT GTT TAC GAG TGC AAT AGC ATG TGC AAC TGC AAT AAA ACA TGC CCA [2184]

#OsSDG706 --- --- --- GTT ATT TTG CAG GAA GGA TAT CCT ATA TAT GAG TGC AAT TCA TCA TGC ACT TGT GAT GCC TCC TGT CAG [2184]

#SmSDG3 --- --- --- ATC GTT CTT CAA GAG GGT TAC CTC GTT TAT GAG TGC AAT ACG AGT TGT ATG TGC TCC GAG GAG TGT CCG [2184]

#PpSDG4 --- --- --- ATC ATT CTT GAT GTT GGA TAC ATG GTT TAT GAA TGC AAT TCT AGT TGT CAG TGT AAA GAT TCT TGC CGC [2184]

#PpSDG5 --- --- TGC GCA TGT GGA GAA GGT AGG TGT GAT TCT GGC CAC TGT GAT CAC GTC TTG ATG TTT GAC AAT GAC AAC GGG [2184]

#AtSDG20 AAG TGT TTA GCT TTC GCG GGG ATG GAA GAG ATA GCT AAC GAA TGT GGG TCG GGT TGT GGA TGC GGG TCC GAT TGT TCG [2184]

#SmSDG4 CAG TTC AAA ACG GTT GGT TTG AAG CCT ACC ATG CTA CGC GAC CTA CGC CAG GTC TGG GAA TTG AAA TTG GGA GTG GCG [2184]

#SmSDG6 --- --- GCT CCA ACT CCC GAC GAT CTT CCA CTG ATC TAC GAG TGC GGC CCT GCT TGC TCG TGC ACC ATC CAA TGC TGC [2184]

#PpSDG9 --- GAC AGT GGT GTG AAT GAA GAA CCG CCA CTT GTT TTG GAG TGT GGA GGA GCA TGT ATC TGC AGT GCA GAT TGT TGC [2184]

#CrSDG1 --- --- GCC ATC GCC CTT GTG GAT GCA GGG ATT GGG GAC GAC ACG GCG ACG CCG CCG GGG TGC GTG CTG GGC TGC GCT [2184]

# Position 15

#AtSDG33 AAC CGA ACT TCT CAG AAG CGT CTA AGA TTC AAT CTT GAG GTT TTC CGC TCT GCA AAG --- --- --- --- --- --- --- [2262]

#PtSDG10 AAC CGC GTC TCT CAG CGT GGA TTG AAG TAC CAA CTT GAG ATC TAT CGT ACA GCA GAC --- --- --- --- --- --- --- [2262]

#OsSDG714 AAT AGA ACA TCT CAG AAA GGT CTG CAG TAT CGC TTG GAG GTA TTT AAG ACA GCT TCA --- --- --- --- --- --- --- [2262]

#SmSDG1 AAC CGA GTG ACG CAG CGT GGC CTA CGT TAC CGC CTT GAG GTG TAC AAA ACC CAG CAC --- --- --- --- --- --- --- [2262]

#PpSDG1 AAT CGA ACA TCT CAA GTG GGG CTT CAA TAT AGG CTT GAG GTG TAC AAA ACT GTT TCA --- --- --- --- --- --- --- [2262]

#AtSDG13 AAC CGA GTG GTG CAA AGA GGA ATG CAC AAC AAA TTA CAG GTG TTT TTC ACG CCA AAT GGG --- --- --- --- --- --- [2262]

#AtSDG18 AAC CGA GTT GTC CAA CAG GGC ATT CAC AAC AAG CTG CAG GTG TTT TTC ACG CCC AAT GGG --- --- --- --- --- --- [2262]

#AtSDG31 AAT CGA GTA GTA CAG AGA GGG ATA AGG TGC CAA CTG CAG GTT TAC TTT ACT CAA GAA GGG --- --- --- --- --- --- [2262]

#PtSDG15 AAT AGA GTC GTG CAG CGA GGT ATA ATG TGC AAG TTG CAG GTT TTC TTT ACT CCT GAA GGA --- --- --- --- --- --- [2262]

#PtSDG18 AAT CGT GTT GTC CAG CGA GGG ATA CAA GTT GCT TTG CAG GTT TTT GCA GCA CCT GAA GGG --- --- --- --- --- --- [2262]

#OsSDG712 AAC CGT GTG GTG CAG CGA GGC ATC ACA CGC CAT TTA CAG GTG TTC TTA ACC CCT GAA AAA --- --- --- --- --- --- [2262]

#PpSDG7 AAT CGA GTG GTA CAA CGT GGT ATC AGT CGT AAG CTG GAG GTG TAT ATG ACG CCT GAA GGC --- --- --- --- --- --- [2262]

#PpSDG8 AAC CGG ATC GTG CAA CGA GGC ATC ACC GCG AGA CTG CAG ATG CCA GTC GAG CTT GAA TCG GGG ATG GGT CAG GTC TTC [2262]

#AtSDG6 AAT CGG GTT TTG CAG AAT GGA ATT CGC GCG AAA CTA GAA GTT TTT AGA ACT GAA AGC --- --- --- --- --- --- --- [2262]

#PtSDG11 AAT AGA GTT TTG CAG AAT GGA ATA CGA GTG AAA CTG GAA GTC TTC AAA ACA GAT AAT --- --- --- --- --- --- --- [2262]

#OsSDG706 AAT AAA GTA CTG CAG AGA GGA CTG CTG GTA AAG TTG GAA GTC TTC AGA ACT GAG AAT --- --- --- --- --- --- --- [2262]

#SmSDG3 AAT CGT GTT CTT CAG AGA GGT GTA AAA GTT AAA CTG GAG GTT TTC AAG ACG AGG CAC --- --- --- --- --- --- --- [2262]

#PpSDG4 AAT CGC GTG CTA CAG AAG GGT GTT CGT TTG AAA CTT GAA GTT TTC AAA AGC CGA CAC --- --- --- --- --- --- --- [2262]

#PpSDG5 GAG GCC TGC GAC AAG TCG GGA GTA GCT ATA AAA GGT CGA TTC CCT TAT GAT GCT CAA GGC --- --- --- --- --- --- [2262]

#AtSDG20 AAC CGG GTT ACG CAG AAG GGG GTT TCG GTT AGT TTG AAG ATT GTG AGA GAT GAG AAG --- --- --- --- --- --- --- [2262]

#SmSDG4 AAT TGG TGT ATT GAT GAG GAT GTG GGC ATC TCT AAG TCA CTG TTT GCA TGG TGG AAG CTG AAT --- --- --- --- --- [2262]

#SmSDG6 CAC AGG CTC TCC CAG CGC GGC GCC TCC GCC GAG CTC AAG GTT GTG AGG CAT CCA ACC --- --- --- --- --- --- --- [2262]

#PpSDG9 CAT CGA GTT ACA CAG CAA GGT CTC TCT GCC CGC GTC GTT GTC ACT CGT CAA CGT TTT ACG --- --- --- --- --- --- [2262]

#CrSDG1 GCC CGG CTA ACG CAA CAC GGG CTG GCG GCG CGT GTG CGT CTG TCG TGG GTG CCC GGC --- --- --- --- --- --- --- [2262]

#AtSDG33 --- --- --- --- AAG GGT TGG GCA GTT AGA TCA TGG GAG TAC ATA CCA GCT GGT TCA CCA GTA TGT GAG TAC ATA GGA [2340]

#PtSDG10 --- --- --- --- AAA GGA TGG GCT GTT AGG TCT TGG GAT GTA ATT CCT TCT GGT GCT CCA GTT TGT GAA TAC TTT GGA [2340]

#OsSDG714 --- --- --- --- AAA GGC TGG GGT GTC AGG ACT TGG GAT ACT ATC CTC CCT GGG GCT CCC ATC TGT GAG TAC ACT GGT [2340]

#SmSDG1 --- --- --- --- AAA GGA TGG GCT GTT AGA TCA TGG GAT TCC ATC CCT GCT GGA GCG CCC GTC TGT GAA TAC TTT GGC [2340]

#PpSDG1 --- --- --- --- AAG GGG TGG GCT TGC CGA TCA TGG GAT TTT ATT CCA GCG GGA GCA CCA ATC TGC GAG TAC TTC GGT [2340]

#AtSDG13 --- --- --- --- AAA GGC TGG GGA CTT AGG ACT TTA GAG AAA CTG CCT AAA GGG GCA TTT ATT TGT GAG TAC ATT GGA [2340]

#AtSDG18 --- --- --- --- AGA GGC TGG GGA CTC AGA ACT CTA GAA AAA CTG CCT AAA GGA GCA TTT GTC TGT GAG CTT GCT GGA [2340]

#AtSDG31 --- --- --- --- AAA GGA TGG GGT CTT AGA ACA CTG CAA GAC TTG CCC AAA GGA ACC TTT ATC TGT GAA TAC ATT GGT [2340]

#PtSDG15 --- --- --- --- AAG GGG TGG GGT CTC AGA ACT CTA GAA TTG CTA CCA AAA GGT ACA TTT GTA TGT GAG TAT GTA GGG [2340]

#PtSDG18 --- --- --- --- AAA GGG TGG GGT GTT CAA TCT GTG AAT GCC CTG AAG AAA GGC ACT TTC ATT TGT GAG TAT GTA GGC [2340]

#OsSDG712 --- --- --- --- AAA GGA TGG GGA TTG CGC AGT ACT GAG AAA CTT CCT CGA GGT GCT TTT GTT TGT GAG TAT GTT GGT [2340]

#PpSDG7 --- --- --- --- AAG GGG TGG GGC ATC AGG ACT TTG GAG GAT CTT CCG GCC GGT GCG TTT GTT TTT GAA TAT GTA GGC [2340]

#PpSDG8 TGG ACT GGC GGA AAA GGC TGG GGT GTG CGA GCA TTG GAT TAT CTA CCG GCT GGC ACG TTT GTA TGC GAG TAC GTG GGA [2340]

# Position 16

#AtSDG6 --- --- --- --- AAG GGA TGG GGA TTG CGA GCT TGT GAA CAT ATA CTG CGT GGC ACA TTT GTT TGC GAA TAC ATC GGG [2340]

#PtSDG11 --- --- --- --- AAA GGT TGG GCT GTC AGG GCA GGT GAA CCA ATC CTA CGT GGT ACA TTT ATA TGT GAG TAC ACT GGA [2340]

#OsSDG706 --- --- --- --- AAG GGT TGG GCT GTT AGA GCC GCA GAA CCT ATT CCA CAG GGC ACA TTT GTA TGT GAG TAC ATT GGA [2340]

#SmSDG3 --- --- --- --- AAG GGA TGG GCT GTG AGG GCC GCT CAA AAC ATC TCC CGT GGA ACG TTT GTT TGC GAG TAC CTT GGT [2340]

#PpSDG4 --- --- --- --- AAG GGA TGG GGT GTG CGA GCA GCG GAG CCC ATA TCA AGG GGT ACA TTT GTG TGT GAG TAT ATA GGG [2340]

#PpSDG5 CGA ATC ATT CTT GAG GGT TGG GCT GTT CGG AGT GCA CAG CCG ATT CCT TCT GGG ACC TTC GTG TGT GAG TAT ATA GGC [2340]

# Position 17

#AtSDG20 --- --- --- --- AAA GGT TGG TGC TTG TAC GCT GAC CAG CTT ATC AAG CAA GGC CAA TTC ATC TGT GAA TAT GCA GGT [2340]

#SmSDG4 --- --- GAG AAG ATA GGC TAC CTG GTC GCA GAC TCG TCT GGG AAC AAG GCA ACT GGG CTT GTG AAG CAG GCC GGG TCC [2340]

#SmSDG6 --- --- --- --- AAA GGC TGG AGC TTA CAT GCA GCG CAG GAC ATC AAA CCG GGA GCT TTC ATC TGC GAG TAC GCT GGA [2340]

#PpSDG9 --- --- --- --- --- GGT TGG GGT TTA CAC GCT GCA CAG CAC ATC AGC AAA GGC TCA TTC GTC TGT GAA TAC GCA GGT [2340]

#CrSDG1 --- --- --- --- AAG GGA TGG GCC GCG TTT GCC GCC GAG CCC CTG CCC GCC GGC GCC TTC GTG TGC CGC TAT GAA GGG [2340]

#AtSDG33 GTT GTC AGG AGA ACT GCT GAT --- GTG GAT ACT ATC TCT GAC AAT GAA TAC ATA TTT --- --- --- --- --- --- --- [2418]

#PtSDG10 ATT CTT AGG AGG AAT GAC GAG --- TTG GAT AAC GTC TCT GGG AAT GAA TTT ATA TTT --- --- --- --- --- --- --- [2418]

#OsSDG714 GTG CTG AGG AGG ACT GAA GAA --- GTA GAT GGT TTG CTG CAG AAC AAT TAC ATA TTT --- --- --- --- --- --- --- [2418]

#SmSDG1 AAA GTT ATA AAG TCT GAC AGT --- --- CTC GAC GTA AAG AGC GAC GTC TAT TTG TTT --- --- --- --- --- --- --- [2418]

#PpSDG1 ACT TTG AGA CGG AAT GAT GAG AAC TTG GAA AGT ATG CTG GAT AAC AGT TAT ATT TTT --- --- --- --- --- --- --- [2418]

#AtSDG13 GAG ATA TTG ACA ATT CCG GAG TTA TAC CAA CGT AGC --- --- --- --- --- --- --- --- --- --- --- --- --- --- [2418]

#AtSDG18 GAG ATA TTG ACC ATT CCA GAG TTG TTC CAA CGC --- --- --- --- --- --- --- --- --- --- --- --- --- --- --- [2418]

#AtSDG31 GAA ATA TTG ACC AAC ACG GAG TTA TAC GAT CGG AAT GTT AGG TCT AGT --- --- --- --- --- --- --- --- --- --- [2418]

#PtSDG15 GAG ATC CTA ACC AAC AAG GAG TTC TAC GAG AGG AAG ATG CAA AGG GCA ACT AGC AAC --- --- --- --- --- --- --- [2418]

#PtSDG18 GAA ATT GTG ACG AAT CAG GAA CTA TAC GAG CGA AAC AAT GAA AGG GCT GCT AAA --- --- --- --- --- --- --- --- [2418]

#OsSDG712 GAA ATA TTA ACG AAC ATT GAG TTG TAT GAC CGT ACA ATT CAA AAG ACT GGT --- --- --- --- --- --- --- --- --- [2418]

#PpSDG7 GAA ATC CTC ACA AAT ACT GAG ATG TGG GAG CGA AAC AAT GAG ATC ATT AGA AAT --- --- --- --- --- --- --- --- [2418]

# Position 19

#PpSDG8 GAA ATA CTG ACC AAC ACA GAG ATG TGG TTT CGC AAC AAC GAG AGC --- --- --- CAC --- --- --- --- --- --- --- [2418]

#AtSDG6 GAG GTT CTT GAT CAG CAA GAA --- --- GCA AAC AAG AGG CGA AAC --- --- CAG TAT --- --- --- --- --- --- --- [2418]

#PtSDG11 GAG ATT TTA AAT GAA CAG GAG --- --- GCA AGC AAT AGG CGT GAC --- --- AGG TAT --- --- --- --- --- --- --- [2418]

#OsSDG706 GAG GTT TTG AAG ATG AAG GAT GAT GGG GCC ATA AGA CAT GTA GAA --- --- AGG GAG --- --- --- --- --- --- --- [2418]

#SmSDG3 GAA GTA CTG AAC GAT CAA GAA --- --- GCA AAT CGG CGA GGA GAA --- --- AGT --- --- --- --- --- --- --- --- [2418]

#PpSDG4 GAG GTT CTT AAT GAC AAG GAA --- --- GCA AAT GAA AGA GGG AAG --- --- AGA TAT --- --- --- --- --- --- --- [2418]

# Position 18

#PpSDG5 GAA GTA GTC AAT GAT CGG GAG GCT AAC CAG AGA GGC GTT TTC CTG CCC ATT TTC AGA GGC TTT ACA CTT TTC TTT TAC [2418]

#AtSDG20 GAG CTA TTG ACA ACA GAT GAA GCA CGT AGA CGT CAA AAC ATT TAC GAC AAA CTC --- --- --- --- --- --- --- --- [2418]

#SmSDG4 CAA CTT GCT CAC ACA ACT GAT GGT ATT GTG TTT GAT GGT GCA ACA TAC ATT TTC ATG GGC --- --- --- --- --- --- [2418]

#SmSDG6 GAG CTC TTG ACG ACC AAG GAA GCT CGG AAA AGG CAC CAA ACT TAC GAC --- --- --- --- --- --- --- --- --- --- [2418]

#PpSDG9 GAA CTG CTA ACA ACT GTG CAA TCT CGA GAG CGT CAG AGC TTA TAT GAT GCC GGA AAT --- --- --- --- --- --- --- [2418]

#CrSDG1 GAG CTG CTG CGC AGC GGG GAG GCG GAG CGG CGG CTA CGG CAC GTG TAC GAC TGC AGC --- --- --- --- --- --- --- [2418]

#AtSDG33 --- --- --- --- --- --- --- --- --- --- --- --- GAG ATT GAC TGC CAA CAG ACA ATG CAA GGT CTT GGT GGA AGA [2496]

#PtSDG10 --- --- --- --- --- --- --- --- --- --- --- --- GAT ATT GAC TGC TGG CAT ACA ATG AAT GAA ATA GGG GGA AGA [2496]

#OsSDG714 --- --- --- --- --- --- --- --- --- --- --- --- GAT ATT GAT TGT CTT CAA ACT ATG AAG GGT CTG GAT GGA AGA [2496]

#SmSDG1 --- --- --- --- --- --- --- --- --- --- --- --- GAT CTG GAT TGC ATT CAA ACC ATG CGA GGA GTT GAC GGC AGA [2496]

#PpSDG1 --- --- --- --- --- --- --- --- --- --- --- --- GAG CTC GAT CTA CTT CAA ACT ATG CAA GGC ATG GAA GGG CGC [2496]

#AtSDG13 --- --- --- --- --- --- --- --- --- --- --- --- TTT GAA GAT AAA CCC ACT TTG CCT GTG ATA TTG GAT GCA CAT [2496]

#AtSDG18 --- --- --- --- --- --- --- --- --- --- --- --- ATC TCT GAC AGG CCT ACT TCT CCA GTA ATA TTG GAT GCG TAC [2496]

#AtSDG31 --- --- --- --- --- --- --- --- --- --- --- --- --- AGT GAA CGA CAT ACA TAT CCT GTA ACT CTG GAT GCA GAC [2496]

#PtSDG15 --- --- --- --- --- --- --- --- --- --- --- --- AAA ACT GAA AAG CAT GCC TAT CCA GCT GTA CTT GAT GCA GAT [2496]

#PtSDG18 --- --- --- --- --- --- --- --- --- --- --- --- --- AAG GAG AGG CAT ACT TAT CCA GTG CTG CTG GAT GCA GAC [2496]

#OsSDG712 --- --- --- --- --- --- --- --- --- --- --- --- --- AAA GCA AAG CAC ACA TAC CCA TTG TTA CTT GAT GCC GAC [2496]

#PpSDG7 --- --- --- --- --- --- --- --- --- --- --- --- GGT GAG GGA CGT CAC ACT TAC CCT GTG GCC TTG GAT GGC GAC [2496]

#PpSDG8 --- --- --- --- --- --- --- --- --- --- --- --- CGA TCA GCC AAA CAT CAC TTC TCC CTG AAT CTG GAC GCG GAT [2496]

#AtSDG6 --- --- --- --- --- --- --- --- --- --- --- --- GGA GAT TGC AGC TAC ATA CTG GAC ATT GAC GCT AAT ATC AAC [2496]

#PtSDG11 --- --- --- --- --- --- --- --- --- --- --- --- GGC AAA GAA GTT TGC AGC TAT ATG TAT AAA ATT GAT GCT CAT [2496]

#OsSDG706 --- --- --- --- --- --- --- --- --- --- --- --- GCA AAA AGT GGT AGC AGC TAC TTG TTT GAA ATC ACT TCT CAG [2496]

#SmSDG3 --- --- --- --- --- --- --- --- --- --- --- --- --- --- --- --- --- --- --- --- --- --- --- --- --- --- [2496]

#PpSDG4 --- --- --- --- --- --- --- --- --- --- --- --- GAT CAA GTG GGC TGC AGT TAT TTA TAC AAC ATT GAT GCA CAT [2496]

#PpSDG5 GCT CAA GAT TTT CTT AAA TTG TTC ATA TTC AGG TAT GAC CAA GAT GGT TGC AGT TAC CTT TAT GAT ATC GAT GCA CAC [2496]

#AtSDG20 --- --- --- --- --- --- --- --- --- --- --- --- --- AGA TCA ACA CAA TCC TTC GCT TCA GCT CTT TTG GTC GTA [2496]

#SmSDG4 --- --- --- --- --- --- --- --- --- --- ACC ACG CCC TCT CTC TCT GGG CAG ACT GAT TTT GCT GTT CAT GCT TGG [2496]

#SmSDG6 --- --- --- --- --- --- --- --- --- --- --- --- --- CAA TCC CCG AGA --- --- GCT ACG AGC CTT TTG GTG GTG [2496]

#PpSDG9 --- --- --- --- --- --- --- --- --- --- --- --- ACC TCA TGT GGT TCT GCG TTA TTG GTT GTG CGA GAG TAC ATG [2496]

#CrSDG1 --- --- --- --- --- --- --- --- --- --- --- --- AGT AGC AGT CGT TAT GGT AGC AGC AGT GGC AGT AGC AGC CGC [2496]

# Position 20

#AtSDG33 CAG AGA AGA CTA AGA --- GAT GTT GCT GTA CCA ATG AAT AAT --- --- --- --- --- --- --- --- --- GGA GTC AGT [2574]

#PtSDG10 GAG AGG CGA CAA GGT GAT GGG TCT GCA CCT GCA ATT GAT CCT --- --- --- --- --- --- --- --- CTG AAG AAA GTA [2574]

#OsSDG714 GAG AAA AGG GCT GGC TCT GAC ATG CAT CTG CCG TCT CTT CAT --- --- --- --- --- --- --- --- --- GCG GAG AAT [2574]

#SmSDG1 CAG AGA CGG TGG GGA --- GAC TTG AAC AAG TTT TTG GAC TAC CAG AAT GGG AAA GTT AGC TGC GAG TCC AGG GAC GCT [2574]

#PpSDG1 CAG AAA AGG TTT GGC --- GAT GTA ATG CCG GAG CTT AGT --- --- --- --- --- --- --- --- --- --- --- GAT GAA [2574]

#AtSDG13 TGG GGT TCT GAA GAA --- --- --- --- --- --- --- --- --- --- --- --- --- --- --- --- --- --- --- --- --- [2574]

#AtSDG18 TGG GGT TCT GAA GAC --- --- --- --- --- --- --- --- --- --- --- --- --- --- --- --- --- --- --- --- --- [2574]

#AtSDG31 TGG GGT TCT GAA AAG --- --- --- --- --- --- --- --- --- --- --- --- --- --- --- --- --- --- --- --- --- [2574]

#PtSDG15 TGG TGC TTG AAA GGA --- --- --- --- --- --- --- --- --- --- --- --- --- --- --- --- --- --- --- --- --- [2574]

#PtSDG18 TGG GGC TCT GAA AGG --- --- --- --- --- --- --- --- --- --- --- --- --- --- --- --- --- --- --- --- --- [2574]

#OsSDG712 TGG GGT ACT GAA GGT --- --- --- --- --- --- --- --- --- --- --- --- --- --- --- --- --- --- --- --- --- [2574]

#PpSDG7 TGG GGT TCT GAA GCC --- --- --- --- --- --- --- --- --- --- --- --- --- --- --- --- --- --- --- --- --- [2574]

#PpSDG8 TGG TGC TCC GAA CGA --- --- --- --- --- --- --- --- --- --- --- --- --- --- --- --- --- --- --- --- --- [2574]

#AtSDG6 GAC ATA GGT --- --- --- --- --- --- --- --- --- --- --- --- --- --- --- --- --- --- --- --- --- --- --- [2574]

#PtSDG11 ACC AAT GAT ATG AGC --- --- --- --- --- --- --- --- --- --- --- --- --- --- --- --- --- --- --- --- --- [2574]

#OsSDG706 ATT GAC AGG GAA AGA --- --- --- --- --- --- --- --- --- --- --- --- --- --- --- --- --- --- --- --- --- [2574]

#SmSDG3 --- --- --- --- --- --- --- --- --- --- --- --- --- --- --- --- --- --- --- --- --- --- --- --- --- --- [2574]

#PpSDG4 CTA GAT GTG ATT GGT --- --- --- --- --- --- --- --- --- --- --- --- --- --- --- --- --- --- --- --- --- [2574]

#PpSDG5 CTG GAC ATG TCG ATC TCT --- --- --- --- --- --- --- --- --- --- --- --- --- --- --- --- --- --- --- --- [2574]

#AtSDG20 CGC GAA CAC CTC CCT --- --- --- --- --- --- --- --- --- --- --- --- --- --- --- --- --- --- --- --- --- [2574]

#SmSDG4 GTG AAG GTC CCA CCC CGC --- --- --- --- --- --- --- --- --- --- --- --- --- --- --- --- --- --- --- --- [2574]

#SmSDG6 AGA GAA CAT CTT CCA --- --- --- --- --- --- --- --- --- --- --- --- --- --- --- --- --- --- --- --- --- [2574]

#PpSDG9 CCC TCA GGA GAG GCG TGT GTT CGG --- --- --- --- --- --- --- --- --- --- --- --- --- --- --- --- --- --- [2574]

#CrSDG1 AGT AGC AGT GGC AGT GGC AGC ACG GGC GCT GGC TTC TAT AAG --- --- --- --- --- --- --- --- --- --- GAG GAG [2574]

#AtSDG33 CAG AGC AGT GAA GAT GAG AAT GCG CCA GAG TTC TGC ATT GAT GCT GGT TCA ACA GGA AAC TTT GCT AGG TTT ATA AAT [2652]

#PtSDG10 GAT GTC AAG ATG GAT GAA AGT GAA TCT GAG TTT TGC ATA GAT GCG GGC TCT TAT GGT AAT GTT ACC AGA TTT ATT AAT [2652]

#OsSDG714 GAT TCA --- --- GAT CCA CCT GCA CCA GAG TAT TGC ATT GAT GCT GGC TCT ATT GGC AAC TTT GCA AGG TTT ATA AAC [2652]

#SmSDG1 GAG GAT GCG GAA CAC CAT GGA CAA GCG GAG TTT TGC CTG GAC GGA GGC GAG TGT GGA GCA GTA GCG CGT TTT ATC AAT [2652]

#PpSDG1 GAC GAC TTG ATG ATG CAA GAT GCT CCC GCT TAT GTT CTT GAT GCT GGA AAG AAT GGA AGT GTT TCA AGA TTT CTT AAT [2652]

#AtSDG13 --- --- --- AGG TTA GAG GGT GAC AAA GCT CTG TGT CTT GAT GGG ATG TTT TAT GGA AAT ATC TCA AGG TTC CTT AAC [2652]

#AtSDG18 --- --- --- ATT TCA GGG GAT GAC AAA GCA CTT TCT CTT GAA GGA ACG CAC TAT GGA AAT ATT TCC AGG TTC ATC AAT [2652]

#AtSDG31 --- --- --- GAT CTA AAA GAT GAA GAA GCT CTC TGC CTG GAT GCC ACA ATC TGT GGA AAT GTC GCA AGG TTT ATC AAT [2652]

#PtSDG15 --- --- --- GTT GTA AAT GAT GAA GAA GCT CTG TGC TTG GAT GCG ACA TTT TAT GGA AAT GTT GCT AGG TTT ATC AAC [2652]

#PtSDG18 --- --- --- ATA CTG GAG GAT GAG GAG GCC CTT TGC TTG GAC GCA ACA GAA TTT GGA AAC ATT GGC AGG TTT ATC AAT [2652]

#OsSDG712 --- --- --- GTT CTT AAG GAT GAG GAA GCC CTT TGT CTA GAT GCC ACG TTT TAT GGT AAC GTC GCA AGA TTT ATA AAC [2652]

#PpSDG7 --- --- --- AAC CTG AAG GAT GAG GAG GCA CTT TGC TTG GAT GCC ACA TAC TTT GGC AAT GTT GCA AGA TTT CTT AAT [2652]

#PpSDG8 --- --- --- TAC CTC AAG GAT GAG GAA GCT CTT TGT TTG GAT GGT ACT TGC TAT GGG AAT GTC GCC CGT TTC ATC AAC [2652]

#AtSDG6 AGA TTG ATG GAA GAA GAG CTC GAT TAT GCT ATT GAT GCT ACT ACT CAT GGC AAC ATC TCT CGA TTC ATC AAT CAC --- [2652]

#PtSDG11 --- --- AGA ATG GTT GAA GGA CAG GCC CAT TAT TTT ATT GAT GCT ACA AAG TAT GGG AAT GTT TCA CGG TTC ATC AAT [2652]

#OsSDG706 --- --- GTT CAG ACT ACA GGG ACT ACT GCA TAC GTG ATT GAT GCC ACA AGA TAT GGC AAC GTA TCC CGT TTT ATT AAT [2652]

#SmSDG3 --- --- --- --- --- --- --- --- --- --- --- --- --- --- --- --- --- --- --- --- --- --- --- --- --- --- [2652]

#PpSDG4 --- --- TCA AAA TCT GTT TCA AAA CCT --- TTT GTA ATA GAT GCA ACC AAA TAT GGA AAT GTT GCT CGA TTT ATT AAT [2652]

#PpSDG5 --- --- --- --- CGA GCT GGT GCA AAA CCG TTT GTG ATT GAT GCT ACT AAG CAT GGA AAT GTC GCT CGC TTC ATC AAC [2652]

#AtSDG20 --- --- --- TCA GGA CAA GCT TGT TTA AGG ATA AAC ATC GAC GCC ACA AGA ATT GGG AAC GTT GCT AGA TTC ATC AAC [2652]

#SmSDG4 --- --- ATG AAG CAG GAA CTG TGC GTC ATC CAG CAA CGC TCT GAT CAA ATC TAT GGT GAG TGG TGG CTA TGG ATC CTC [2652]

#SmSDG6 --- --- --- AAG GGC GAC GCA TGC CTC CGA TTC AAC ATC GAT GCC ACC AAC GTA GGC AAC ATT GCC AGG TTC ATC AAT [2652]

# Position 21

#PpSDG9 --- --- --- ATC AAC GTA GAT GCT ACT AAA ATG CTA CAT GAG CGC CCA GCT AGC TCT GAA CTA TCT CGA TCT AGA CCA [2652]

#CrSDG1 GGA GAG GAG GTG GAG CGG AAG GAG GTG GGG CGT GCG ACG GAT GCC TGC GTC AGA GGG CAA GGC GAC CGC GGC GGC GGC [2652]

#AtSDG33 CAC --- --- --- --- --- --- --- --- --- --- --- --- --- --- --- --- --- --- --- --- --- --- --- --- --- [2730]

#PtSDG10 CAT --- --- --- --- --- --- --- --- --- --- --- --- --- --- --- --- --- --- --- --- --- --- --- --- --- [2730]

#OsSDG714 CAC --- --- --- --- --- --- --- --- --- --- --- --- --- --- --- --- --- --- --- --- --- --- --- --- --- [2730]

#SmSDG1 CAC --- --- --- --- --- --- --- --- --- --- --- --- --- --- --- --- --- --- --- --- --- --- --- --- --- [2730]

#PpSDG1 CAC --- --- --- --- --- --- --- --- --- --- --- --- --- --- --- --- --- --- --- --- --- --- --- --- --- [2730]

# Position 23

#AtSDG13 CAC AGA --- --- --- --- --- --- --- --- --- --- --- --- --- --- --- --- --- --- --- --- --- --- --- --- [2730]

#AtSDG18 CAC AGA --- --- --- --- --- --- --- --- --- --- --- --- --- --- --- --- --- --- --- --- --- --- --- --- [2730]

#AtSDG31 CAC AGA --- --- --- --- --- --- --- --- --- --- --- --- --- --- --- --- --- --- --- --- --- --- --- --- [2730]

#PtSDG15 CAC AGA --- --- --- --- --- --- --- --- --- --- --- --- --- --- --- --- --- --- --- --- --- --- --- --- [2730]

#PtSDG18 CAC AGG --- --- --- --- --- --- --- --- --- --- --- --- --- --- --- --- --- --- --- --- --- --- --- --- [2730]

#OsSDG712 CAC AGG --- --- --- --- --- --- --- --- --- --- --- --- --- --- --- --- --- --- --- --- --- --- --- --- [2730]

#PpSDG7 CAC AGG --- --- --- --- --- --- --- --- --- --- --- --- --- --- --- --- --- --- --- --- --- --- --- --- [2730]

#PpSDG8 CAT GGC --- --- --- --- --- --- --- --- --- --- --- --- --- --- --- --- --- --- --- --- --- --- --- --- [2730]

#AtSDG6 --- --- --- --- --- --- --- --- --- --- --- --- --- --- --- --- --- --- --- --- --- --- --- --- --- --- [2730]

#PtSDG11 CAC AGT TTG AGA ATT TTG CTT CTT GGA GAC AAA TGT ATC AAT GTG AAC CAA TTA AAA TTT CCT CAT ACT CGA TTG AAC [2730]

#OsSDG706 CAT --- --- --- --- --- --- --- --- --- --- --- --- --- --- --- --- --- --- --- --- --- --- --- --- --- [2730]

#SmSDG3 --- --- --- --- --- --- --- --- --- --- --- --- --- --- --- --- --- --- --- --- --- --- --- --- --- --- [2730]

#PpSDG4 CAT --- --- --- --- --- --- --- --- --- --- --- --- --- --- --- --- --- --- --- --- --- --- --- --- --- [2730]

# Position 22

#PpSDG5 CAT AGC --- --- --- --- --- --- --- --- --- --- --- --- --- --- --- --- --- --- --- --- --- --- --- --- [2730]

#AtSDG20 CAT TCT --- --- --- --- --- --- --- --- --- --- --- --- --- --- --- --- --- --- --- --- --- --- --- --- [2730]

#SmSDG4 AAT GGC AAT --- --- --- --- --- --- --- --- --- --- --- --- --- --- --- --- --- --- --- --- --- --- --- [2730]

#SmSDG6 CAC TCC --- --- --- --- --- --- --- --- --- --- --- --- --- --- --- --- --- --- --- --- --- --- --- --- [2730]

#PpSDG9 CTG GGC --- --- --- --- --- --- --- --- --- --- --- --- --- --- --- --- --- --- --- --- --- --- --- --- [2730]

#CrSDG1 GGC GCG GGC --- --- --- --- --- --- --- --- --- --- --- --- --- --- --- --- --- --- --- --- --- --- --- [2730]

#AtSDG33 --- --- --- --- --- --- --- --- --- --- --- --- --- --- --- --- --- --- --- --- AGT TGT GAA CCA AAC CTA [2808]

#PtSDG10 --- --- --- --- --- --- --- --- --- --- --- --- --- --- --- --- --- --- --- --- AGT TGT CAG CCC AAC CTA [2808]

#OsSDG714 --- --- --- --- --- --- --- --- --- --- --- --- --- --- --- --- --- --- --- --- AGC TGC GAG CCT AAC CTT [2808]

#SmSDG1 --- --- --- --- --- --- --- --- --- --- --- --- --- --- --- --- --- --- --- --- AGC TGC GAG CCA AAC CTC [2808]

#PpSDG1 --- --- --- --- --- --- --- --- --- --- --- --- --- --- --- --- --- --- --- --- AGC TGT GAA CCT AAT GTG [2808]

#AtSDG13 --- --- --- --- --- --- --- --- --- --- --- --- --- --- --- --- --- --- --- --- TGT CTT GAT GCA AAC TTG [2808]

#AtSDG18 --- --- --- --- --- --- --- --- --- --- --- --- --- --- --- --- --- --- --- --- TGC TTA GAT GCA AAT TTG [2808]

#AtSDG31 --- --- --- --- --- --- --- --- --- --- --- --- --- --- --- --- --- --- --- --- TGC GAG GAT GCA AAC ATG [2808]

#PtSDG15 --- --- --- --- --- --- --- --- --- --- --- --- --- --- --- --- --- --- --- --- TGT CTC GAT GCT AAC ATG [2808]

#PtSDG18 --- --- --- --- --- --- --- --- --- --- --- --- --- --- --- --- --- --- --- --- TGT TAT GAT TCT AAC TTG [2808]

#OsSDG712 --- --- --- --- --- --- --- --- --- --- --- --- --- --- --- --- --- --- --- --- TGC TTT GAT GCT AAT ATT [2808]

#PpSDG7 --- --- --- --- --- --- --- --- --- --- --- --- --- --- --- --- --- --- --- --- TGC TTA GAT GCA AAC CTT [2808]

#PpSDG8 --- --- --- --- --- --- --- --- --- --- --- --- --- --- --- --- --- GAG TAC CTT TCC AGA TGC CGT TCA GCT [2808]

# Position 25

# Position 24

#AtSDG6 --- --- --- --- --- --- --- --- --- --- --- --- --- --- --- --- --- --- --- --- AGC TGC TCA CCA AAT CTC [2808]

#PtSDG11 CAT TTG ATT TTC TGT AAG GCT GTG CTT GTT GAT TCA CAC TTG ACA TAT GTT CAT GTT GGA AGC TGC ATG CCA AAT CTT [2808]

#OsSDG706 --- --- --- --- --- --- --- --- --- --- --- --- --- --- --- --- --- --- --- --- AGC TGT TCC CCA AAT CTC [2808]

#SmSDG3 --- --- --- --- --- --- --- --- --- --- --- --- --- --- --- --- --- --- --- --- --- TGT TCC CCG AAC CTT [2808]

#PpSDG4 --- --- --- --- --- --- --- --- --- --- --- --- --- --- --- --- --- --- --- --- AGC TGT GAA CCG AAT TTG [2808]

#PpSDG5 --- --- --- --- --- --- --- --- --- --- --- --- --- --- --- --- --- --- --- --- --- TGT GCT CCT AAT CTG [2808]

#AtSDG20 --- --- --- --- --- --- --- --- --- --- --- --- --- --- --- --- --- --- --- --- TGT GAC GGT GGA AAT CTC [2808]

#SmSDG4 --- --- --- --- --- --- --- --- --- --- --- --- --- --- --- CTA TGG TTT ACG GTG TAC AAT GGG GAA TAC CAG [2808]

#SmSDG6 --- --- --- --- --- --- --- --- --- --- --- --- --- --- --- --- --- --- --- --- TGC GAC GGT GGC AAT CTC [2808]

#PpSDG9 --- --- --- --- --- --- --- --- --- --- --- --- --- --- --- --- --- --- --- ATC TCC GTC ACG GAT TGC CTT [2808]

#CrSDG1 --- --- --- --- --- --- --- --- --- --- --- --- --- --- --- --- --- --- --- --- CGG CAA CGA CCG CGG ATA [2808]

#AtSDG33 TTT GTT CAG TGC GTC CTG AGT TCT CAC CAG GAT ATA AGG CTT GCC CGT GTG GTT CTT TTC --- --- --- --- --- --- [2886]

#PtSDG10 TTC GTC CAG TGC ATT TTG AGC ACC CAC CAT GAT ATC CGA CTT GCT CGA ATT GTG CTA TTT --- --- --- --- --- --- [2886]

#OsSDG714 TTT GTC CAG TGT GTC TTG AGC TCC CAT AAT GAT GTT AAG CTG GCA AAG GTG ACG CTC TTT --- --- --- --- --- --- [2886]

#SmSDG1 TTC ATC CAG TGC GTT TTA TCG ACT CAT CAC GAC ATG CGA ATC CCT CGG ATT GTA CTG TTT --- --- --- --- --- --- [2886]

#PpSDG1 TTC ATC CAA TGT GTG TTG AGC CAT CAC AAC GAT GTC ACG ATG CCC CGA ATT GTA ATG TTT --- --- --- --- --- --- [2886]

# Position 26

#AtSDG13 ATC GAG ATT CCA GTT CAA GTC GAG ACT CCG GAT CAG CAT TAT TAT CAT CTT GCA TTC TTC --- --- --- --- --- --- [2886]

#AtSDG18 ATT GAG ATC CCA GTT CAT GCG GAG ACT ACT GAT TCA CAC TAC TAT CAT CTT GCA TTC TTC --- --- --- --- --- --- [2886]

#AtSDG31 ATT GAT ATT CCG ATA GAG ATA GAG ACG CCT GAC AGA CAT TAT TAT CAT ATT GCT TTT TTT --- --- --- --- --- --- [2886]

#PtSDG15 ATC GAG ATC CCG GTG AAG ATT GAG ACC CCT GAT CAT CAT TAT TAT CAT --- --- --- --- --- --- --- --- --- --- [2886]

#PtSDG18 ATT GAG ATT CCA GTG GAA GTG GAG ACT CCG GAT CAT CAC TAT TAT AGA --- --- --- --- --- --- --- --- --- --- [2886]

#OsSDG712 ATA GGA ATA CCT GTT GAG ATC GAG ACG CCC GAC CAC CAT TAT TAC CAT CTG GCG TTC TTC --- --- --- --- --- --- [2886]

#PpSDG7 ATG GAA ATG CCA GTC GAG ATT GAA AGT CCT GAC CGC CAT TAC TAC CAT GTG GCA TTT TTC --- --- --- --- --- --- [2886]

#PpSDG8 GAT CAC CTC CCA AAT CAG GAA AAG GTC GTC ATC GCT ACG TTT AAT GGT CTT GCA TTT TTC --- --- --- --- --- --- [2886]

#AtSDG6 GTT AAT CAC CAA GTT ATT GTG GAA AGC ATG GAG TCC CCG CTC GCT CAT ATC GGT CTA TAC --- --- --- --- --- --- [2886]

#PtSDG11 GTG AAC CAC CAA GTT CTG GTT GAC AGC ATG GAT TCT CAG CGT GCT CAT ATC GGT CTT TAT --- --- --- --- --- --- [2886]

#OsSDG706 AGT ACG CGT TTA GTT TCG GTG GAG AGC AAA GAC TGT CAG CTT GCG CAC ATT GGT TTG TTT --- --- --- --- --- --- [2886]

#SmSDG3 GTA AAC TAT CAG GTG TTA GTG GAG AGC ATG GAC TAT CAA CTC GCG CAC ATT GGA TTA TTT --- --- --- --- --- --- [2886]

#PpSDG4 ATC AAC TAC GAA GTG CTT GTT GAA AGT ATG GAC TGT CAG TTA GCA CAC ATA GGT TTT TTC --- --- --- --- --- --- [2886]

#PpSDG5 ATC AAT TAT GAG GTG CTG GTG GAA AGC ATG GAC TGT CAG CTG GCC CAC ATC GGT TTC TTT GCC AAT --- --- --- --- [2886]

#AtSDG20 TCC ACT GTT CTG TTG AGA AGC --- --- TCA GGA GCG TTG CTT CCT CGG CTC TGT TTC TTT --- --- --- --- --- --- [2886]

#SmSDG4 TTC AGC ATT AAG GGA ACA GTG CCA GTC ACT GAT GGA ACT TGG CAC CAT GTT GCT GCA GTC CGA AAT GGC --- --- --- [2886]

#SmSDG6 CTC TCG TGC CTC GTC CGC TCA --- --- GCA GGC TGC TGC GTC CCC AGG CTG GCT TTC TTC --- --- --- --- --- --- [2886]

#PpSDG9 CTC GAT CAA AGA CGA ATT CTC CTT GGC CCG ACC CCA GAC CAC ATC AAC ATG CCC TTT CTC AGG CAC TCC CAC AAG CTT [2886]

#CrSDG1 GGA GGC GAC GGC GGC ACG AGA GAA GGT GGC GAC AGC GCC GAT GGC GCC GGT GCG GGC GAA GGC --- --- --- --- --- [2886]

# Position 30

#AtSDG33 --- --- --- --- GCA GCT GAC AAC ATT TCC CCA ATG CAG GAG CTC ACT TAC GAC TAT GGA TAT GCG CTT GAT AGC --- [2964]

#PtSDG10 --- --- --- --- GCA GCA GAC GAT ATA CTT CCT ATG CAA GAA CTT ACT TAT GAC TAT GGC TAC GCG CTT GAT AGC --- [2964]

#OsSDG714 --- --- --- --- GCT GCT GAC ACT ATA CTT CCT CTT CAG GAG CTT TCA TAT GAT TAT GGC TAT GTC TTG GAC AGT --- [2964]

#SmSDG1 --- --- --- --- GCA GCT GAC AAC ATA GCA CCA CTT CAG GAG CTA AGC TAC GAC TAT GGC TAT GCA CTC AAC AGT --- [2964]

#PpSDG1 --- --- --- --- GCG GCA GAT AAC ATT CAT CCA TTA GAG GAG TTG TGC TAT GAC TAT GGT TAC GCG ATG GAC AGC --- [2964]

# Position 32

#AtSDG13 --- --- --- --- ACA ACA AGA GAC ATC GAG GCA ATG GAG GAA CTC GCA TGG GAT TAT GGC ATT GAC TTC AAC --- --- [2964]

#AtSDG18 --- --- --- --- ACA ACA AGA GAG ATC GAT GCT ATG GAG GAA CTT ACC TGG GAT TAT GGT GTC CCA TTC AAC --- --- [2964]

#AtSDG31 --- --- --- --- ACC CTA CGA GAC GTG AAG GCC ATG GAT GAG TTG ACA TGG GAT TAC ATG ATA GAC TTC AAT --- --- [2964]

#PtSDG15 --- --- --- --- --- --- --- --- --- --- --- --- --- --- --- --- --- GAT TAT GGC ATT GAT TTT GAT --- --- [2964]

# Position 31

#PtSDG18 --- --- --- --- --- --- --- --- --- --- --- --- --- --- --- --- --- GTA --- --- --- --- --- --- --- --- [2964]

#OsSDG712 --- --- --- --- ACA ACA AGG ATA ATA GAG CCT TTT GAG GAA CTC ACA TGG GAC TAT GGG ATT GAT TTT GAT --- --- [2964]

#PpSDG7 --- --- --- --- ACT AAT CGT CAT GTG AAA GCC AAG GAG GAG CTC ACA TGG GAC TAT GGC ATC GAT TTT GGA --- --- [2964]

# Position 29

#PpSDG8 --- --- --- --- ACC AGC AAA GAT GTA GCA GCA AAT GAA GAG CTC ATA TGG GAC TAT GGG TTG GAT TTC AAC --- --- [2964]

#AtSDG6 --- --- --- --- GCC AGT ATG GAT ATA GCT GCA GGA GAA GAG ATC ACC CGA GAC TAT GGA CGC AGA CCG --- --- --- [2964]

#PtSDG11 --- --- --- --- GCA AGT CAG GAT ATA GCT TTT GGT GAA GAA CTG ACA TAC AAC TAT CGG TAT GAG CTG --- --- --- [2964]

#OsSDG706 --- --- --- --- GCA AAC CAG GAT ATC TTG ATG GGA GAA GAA TTG GCC TAT GAC TAT GGA CAA AAA TTG --- --- --- [2964]

#SmSDG3 --- --- --- --- GCT AGC CGT GAT ATT CTT TGC GGA GAA GAA CTA TCT TAC GAC TAC AGG TAC AAG TTA TTA CCA --- [2964]

# Position 27

#PpSDG4 --- --- --- --- GCC AAT CGC GAT ATT GCA ATT GGG GAG GAG CTT GCA TAT GAC TAT AGG TAT AAA TTG --- --- --- [2964]

#PpSDG5 --- --- --- CGA GAT TGG AAT CAT GAA TCT ATG CCT GAA TAT TTT GTA TGG GAT TCA GCA TAT GTT ACT TAT GAT --- [2964]

#AtSDG20 --- --- --- --- GCA GCA AAG GAC ATA ATC GCA GAG GAG GAG TTA AGT TTC AGT TAT GGA GAC GTA AGT GTG --- --- [2964]

#SmSDG4 --- --- --- --- ACT GTT GGG TCT ATC TAC GTT GAT TCT AAA CTT GAA TCA ACG TCA GTC ACA GAA CGG CTC CAA --- [2964]

#SmSDG6 --- --- --- --- ACG CGC AAA GAG ATC CAG TCA GGC CAA GAA CTT ACT TTC AGC TAT GGT GTC GTC GAA CCG --- --- [2964]

# Position 28

#PpSDG9 CAC GTT CGA GAA AGT GCG GCT CAT CTC TTC TGC CAA ATG GAA CTG GAT CGA TGT GTC AAT CTC ACC GTG AAT GAC --- [2964]

#CrSDG1 --- --- --- --- --- CAT GCG TTG CTG GTT GTG CGC GAG GTG CTG CCC AGC GGA CTG GCC CTG CGG CTC AAC ATT --- [2964]

．．．．．．．．．．．．．．．．．．．．．．．．．．．．．．．．．．．．．．．

#AtSDG33 CAG CTC GCT TGC TAC TGT GGA GCG CTA AAT TGT AGG AAA CGC CTT TAC [3090]

#PtSDG10 CAG TCG CCA TGC TAC TGT GGC ACA GAT GAG TGT CGG GGG CGC TTA TAT [3090]

#OsSDG714 AAG CTG CCT TGC TTC TGT GGT GCA CCG TAC TGC CGG AAG CGA CTT TAT [3090]

#SmSDG1 AAA TTG CCG TGT TAC TGT GGA GCA CTC TCG TGG TTG CGC GAC ATG GCA [3090]

#PpSDG1 GAG ATG GCT TGT CAC TGC GGA GCC GCA TCA TGT CGT AAG CGC ATG TAT [3090]

#AtSDG13 CCC TTT GAT TGT CTG TGT GGT AGC AGA TTC TGC AGG AAC AAG AAG CGA [3090]

#AtSDG18 CCG TTC CAT TGT CAA TGT GGT AGC GAC TTC TGC CGA GTC AGG AAG CAG [3090]

#AtSDG31 GCA TTT AGA TGT TGC TGC GGA AGC GAA TCA TGC AGA GAC AGA AAA ATA [3090]

#PtSDG15 TTA TTC CAT TGC CGA TGT GGC AGC AAG TTC TGC AGG AAC ATG AAA CGT [3090]

#PtSDG18 --- --- --- --- --- --- --- --- --- --- --- --- --- --- --- --- [3090]

#OsSDG712 GCA TTC AAA TGT CAT TGC GGA AGT GAG TTT TGC CGA GAC AAA ACG CGC [3090]

#PpSDG7 GCA TTT CCT TGT TGC TGT GGC AGT GAG TAC TGC AGG GGA AAG ATG GCT [3090]

#PpSDG8 GCT TTT GAA TGC CTT TGT GGA AGT GAC TTC TGT CGA GGG AAA AGC CTT [3090]

#AtSDG6 GAG CAT CCA TGC CAT TGT AAA GCA ACT AAT TGC AGA GGT CTC TTA AGT [3090]

#PtSDG11 --- TAT CCT TGC CAT TGT GGA GCT TCA AAG TGC CGG GGC CGC CTG TAT [3090]

#OsSDG706 --- TGC CCC TGC CAT TGT GGT GCT AAG AAT TGC CGA GGA CGT GTG TAT [3090]

#SmSDG3 GGT TGC CCG TGC CAC TGT GGA TCG TCT GGA TGC CGA GGA AGG TTG TAT [3090]

#PpSDG4 --- TGC CCC TGT TAT TGC GGG GCC CCA AAA TGC CGT GGT CGA CTT TAC [3090]

#PpSDG5 ACA TCT TCT TTT AAG CGG CAC TTA TTA TTA GTC AAA ACT ATG GAT GTT [3090]

#AtSDG20 AAG CTA AAC TGC TCT TGT GGT AGT TCC TGC TGT TTG GGA ACG TTG CCT [3090]

#SmSDG4 --- GAT AGT CAA CCT CAA TTC CAA CCT ACC TGT CTT CAT TGG CTA --- [3090]

#SmSDG6 --- --- GCC TGT TTT TGT GGA ACA AGC CAG TGC CGA GGA ATC CTG CCT [3090]

# Position 33

#PpSDG9 ATT TAT CAA TGC TGG AAT GAC AGC ATG TGT GCC CGC CAG CAA GGA GTT [3090]

#CrSDG1 CGC TTC TTC AAC CAC AGC TGC GAC GGC GGC TGC CTG CTG CCT GTG GTG [3090]
